# Low Temperature Antioxidant Activity QTL Associate with Genomic Regions Involved in Physiological Cold Stress Tolerance Responses in Rice (*Oryza sativa* L.)

**DOI:** 10.3390/genes12111700

**Published:** 2021-10-26

**Authors:** Huy Phan, Michael Schläppi

**Affiliations:** Department of Biological Sciences, Marquette University, Milwaukee, WI 53233, USA; huy.phan@marquette.edu

**Keywords:** anthocyanins, antioxidant activity, catalase, chilling stress, GWAS, membrane integrity, *Oryza sativa* L., ROS, seedling survival, QTL

## Abstract

Boosting cold stress tolerance in crop plants can minimize stress-mediated yield losses. Asian rice (*Oryza sativa* L.), one of the most consumed cereal crops, originated from subtropical regions and is generally sensitive to low temperature environments. Within the two subspecies of rice, *JAPONICA*, and *INDICA*, the cold tolerance potential of its accessions is highly variable and depends on their genetic background. Yet, cold stress tolerance response mechanisms are complex and not well understood. This study utilized 370 accessions from the Rice Diversity Panel 1 (RDP1) to investigate and correlate four cold stress tolerance response phenotypes: membrane damage, seedling survivability, and catalase and anthocyanin antioxidative activity. Most *JAPONICA* accessions, and admixed accessions within *JAPONICA*, had lower membrane damage, higher antioxidative activity, and overall, higher seedling survivability compared to *INDICA* accessions. Genome-wide association study (GWAS) mapping was done using the four traits to find novel quantitative trait loci (QTL), and to validate and fine-map previously identified QTL. A total of 20 QTL associated to two or more traits were uncovered by our study. Gene Ontology (GO) term enrichment analyses satisfying four layers of filtering retrieved three potential pathways: signal transduction, maintenance of plasma membrane and cell wall integrity, and nucleic acids metabolism as general mechanisms of cold stress tolerance responses involving antioxidant activity.

## 1. Introduction

Crop plants are constantly exposed to a broad range of abiotic factors such as extreme temperatures, high salinity, or water deficit, all of which negatively affect growth and development leading to a significant reduction in productivity [1,2]. As the world’s population keeps growing, demand to increase global production of important crops such as rice, wheat, and maize must be met [3,4]. One approach to achieve this goal is to minimize crop yield losses due to environmental stressors. Therefore, it is crucial to increase the cold tolerance of a cold sensitive crop such as rice to better cope with cold temperature exposures during critical physiological stages of development [5,6,7,8].

Low-temperature conditions heavily damage the plant’s ability to grow and develop by disrupting the physical and chemical compositions of the plasma membrane, protein homeostasis, and cellular metabolism [9,10]. Maintaining plasma membrane integrity is one of the vital cold stress response mechanisms in plants [11,12]. Studies have shown that a successful strategy for crop plants to deal with a chilling environment is to quickly alter the physiological characteristics of the cell membrane by changing its lipid composition [13]. This helps to ease the stress of cellular damage caused by the cold, and potentially leads to a successful recovery once the stress ends [11,13].

Various environmental stresses, such as low temperatures, when prolonged, can lead to the over-accumulation of reactive oxygen species (ROS) in crop plants such as rice, wheat, and maize [14,15]. These molecules are highly reactive and toxic to cellular components as they cause damage to proteins, lipids, carbohydrates, and DNA molecules [14,15,16]. Ultimately, this accumulation of ROS damages cell homeostasis, shatters the cellular membrane, and leads to cell death [14,15,16]. However, ROS also act as signaling molecules [15,16,17], but it is important to note that whether ROS act as damaging or signaling factors depends on the delicate equilibrium between ROS production and the plant’s antioxidative ability of scavenging ROS at the proper stage of development [15,17]. Under regular growth conditions, ROS molecules are scavenged by various antioxidative defense mechanisms. In general, these mechanisms can be separated into two groups: enzymatic and non-enzymatic pathways [17].

Antioxidative defense via enzymatic pathways involves a participation of multiple enzymes, most of them members of the peroxidase family [15,17]. They are heme proteins, widely distributed in eukaryotes, and capable of catalyzing the turnover of hydrogen peroxide to water, rendering it harmless [17]. For example, ascorbate peroxidase (APX) uses ascorbate as an electron donor to turnover one molecule of hydrogen peroxide [18]. Likewise, glutathione peroxidase (GPX) uses two molecules of reduced glutathione to break down one molecule of hydrogen peroxide [19]. Catalase (CAT) is a special member of the peroxidase family [20]. This enzyme is encoded mainly by three out of four catalase genes in rice, whose expression levels depend on the stress intensity [21,22]. Unlike other peroxidase enzymes, CAT catalyzes the turnover of two molecules of hydrogen peroxide simultaneously to water and oxygen gas in the cell [20]. Studies in rice have been done to investigate the expression, cloning, and characterization of these CAT genes for abiotic stress tolerance [23,24]. Hence, the ability of CAT turning over hydrogen peroxide may be a proxy for studying the cell’s ability to cope with ROS under low temperature conditions [17,20,21].

The other antioxidative defense in plants is non-enzymatic in which plants utilize a diverse range of phenolic compounds, known as flavonoids, that are primarily derived from phenylalanine [25]. Anthocyanins and its sugar-free counterpart, anthocyanidins, are a major group of flavonoids [25]. It has been shown that low temperatures lead to anthocyanin accumulation, whereas high temperatures decrease anthocyanin content due to disruption of enzymes involved in anthocyanin biosynthesis [26]. The family of anthocyanins contains mainly six members, cyanidins, peonidins, delphinidins, malvidins, pelargonidin, and petunidin [25,26,27]. Cells that are rich in cyanidins have higher turnover rates of ROS, preventing them to cause oxidative damage [28,29], while more peonidins may lead to lignin synthesis, a major component of the plant cell wall [30]. Plants grown under acidic conditions tend to have higher amounts of cyanidins, peonidins, and delphinidins compared to other anthocyanins [27,31,32,33]. Stabilizing the anthocyanin profile with an influx of magnesium ions under low temperature conditions improves rice plant survivability and reduces grain yield loss [33,34]. Together, the plant’s profile of anthocyanin, and its antioxidative activity, may also be a proxy for studying cold tolerance in plants [28,34].

The major purpose of this study was to map new quantitative trait loci (QTL) and associate previously found QTL responsible for cold stress tolerance to antioxidative mechanisms in crop plants using Asian rice (*O. sativa*) as the model organism. Asian rice is an excellent model for this study for the following reasons. First, it is one of the most commonly cultivated cereals for grain production and feeds over half of the world’s population [1,2,11]. Second, Asian rice is the first monocot crop plant whose genome was fully sequenced [35]. Genomics studies revealed that Asian rice has two subspecies: *japonica* and *indica*, and five major subpopulation groups [35,36]. The *japonica* subspecies (hereafter referred to as *JAPONICA*) has three major subgroups: *aromatic* (ARO), *temperate japonica* (TEJ), and *tropical japonica* (TRJ), as well as admixtures within the subspecies (MIX-J) [35,37]. The *indica* subspecies (hereafter referred to as *INDICA*) has two major subgroups: *aus* (AUS) and *indica* (IND), as well as admixtures within the subspecies (MIX-I) [35,37]. Additionally, there are multiple admixtures between the two subspecies (ADMIX) [35,37]. Accessions from subpopulations within the *INDICA* subspecies are generally more-cold sensitive than those from within the *JAPONICA* subspecies, which are in general, relatively cold tolerant [35]. Overall, single nucleotide polymorphism data for more than a thousand accessions are available for genetic mapping of candidate genes that may help improve cold stress tolerance of rice cultivars [35,36,37]. Third, genome-wide association study (GWAS) tools for Asian rice have been developed [38,39] and shown to be successful in mapping not only candidate genes responsible for low temperature responses [40], but also for other abiotic and biotic stressors [41,42,43,44].

## 2. Materials and Methods

### 2.1. Plant Materials

**Figure 1:**
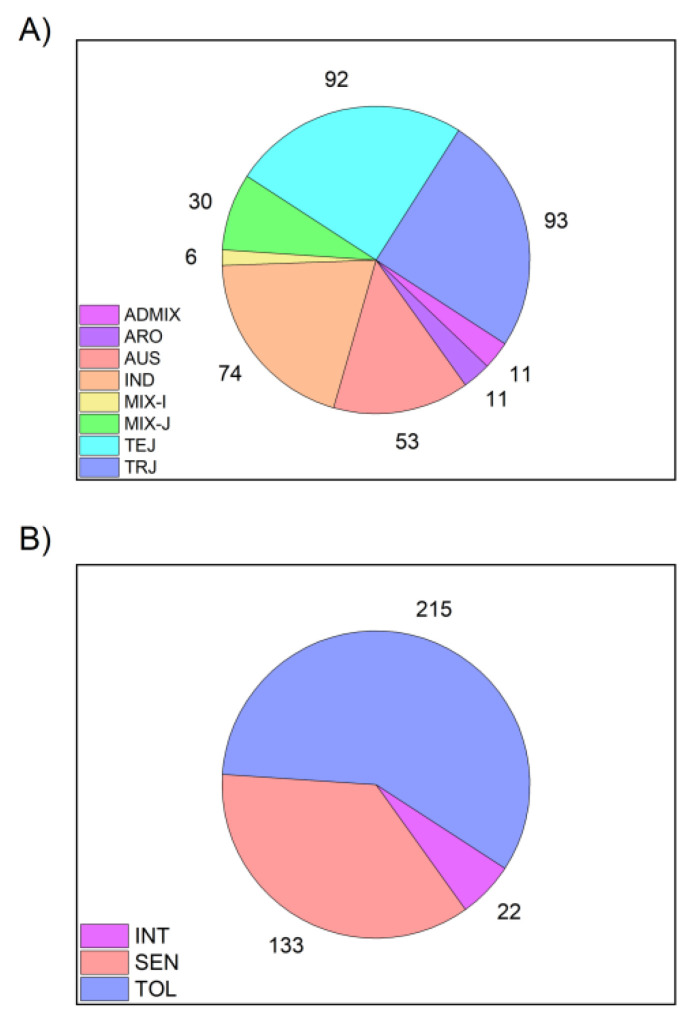
Pie charts of the genetic background composition of the 370 rice accessions used in this study, and the grouping of them into 3 cold tolerance clusters. (**A**) Subpopulation distribution of the 370 accessions. ADMIX: admixes between accessions of the two subspecies *INDICA* (*aus* and *indica*) and *JAPONICA* (*aromatic*, *temperate japonica*, and *tropical japonica*); ARO: *aromatic*; AUS: *aus*; IND: *indica*; MIX-I: admixed *INDICA*; MIX-J: admixed *JAPONICA*; TEJ: *temperate japonica*; TRJ: *tropical japonica*. (**B**) Grouping of the eight subpopulations into 3 cold tolerance clusters. TOL: Cold Tolerant, composed of MIX-J, TEJ, and TRJ; INT: Intermediate Cold Tolerant, composed ADMIX, and ARO; SEN: Cold Sensitive, composed of AUS, IND, and MIX-I (see Figure 2 and Table 1 for details).

Seeds of 370 *O. sativa* accessions from the Rice Diversity Panel 1 (RDP1) were obtained from the Genetic Stocks-Oryza (GSOR) collection located at the USDA-ARS Dale Bumpers National Rice Research Center (USDA-ARS DBNRRC; Stuttgart, AR). The 370 RDP1 accessions utilized in this study represented eight subpopulations (Figure 1): *aus* (53 accessions), *indica* (74), *aromatic* (11), *temperate japonica* (92), *tropical japonica* (93), admixtures of two or more *indica* and *aus* accessions (6), admixtures of two or more *temperate japonica* and *tropical japonica* accessions (30), and admixtures of one or more accessions from *temperate japonica* and *tropical japonica* with one or more accessions from the *indica* and *aus* subpopulations (11). RDP1 accessions were genotyped by McCouch et al. 2016 [38]. Based on previous told tolerance response experiments, we assigned *temperate japonica* and *tropical japonica* accessions, as well as admixtures between them to the Cold Tolerant cluster (TOL), *aromatic* accessions and admixtures between two subspecies to the Intermediate Cold Tolerant cluster (INT), and *aus* and *indica* accessions, as well as admixtures between them to the Cold Sensitive cluster (SEN) [40,44,45].

A smaller subset of 70 accessions was used as warm controls for catalase and anthocyanin activity assays. This subset had representative accession from all eight subpopulation groups: ADMIX (4), ARO (4), AUS (12), IND (12), MIX-I (4), MIX-J (10), TEJ (12), and TRJ (12).

### 2.2. Growth and Stress Conditions

Seeds were germinated in distilled water at 37 °C in the dark for 48 h in petri dishes with 0.1% bleach solution to prevent bacterial contamination. Germinating seeds were transferred into PCR strips after 48 h of germination, placed into pipette tip boxes, and grown hydroponically with deionized water only. Each accession was represented by up to 8 seedlings (one PCR strip) per box in a triplicate randomized block design, i.e., one line per strip, 12 lines per box, 3 boxes per set, 32 sets per trial (including control checks), with a total of 3 trials. Seedlings were grown under 12 h light (approx. 150 µE photon flux)/12 h dark and 28 °C day/25 °C night periods. Distilled water was substituted with quarter strength Murashige-Skoog basal salt medium 10 days after germination. Two cold tolerance checks for growth conditions were cold tolerant *temperate japonica* accession Krasnodarskij 3352, and cold sensitive *aus* accession Carolino 164. The checks were grown in boxes of randomly selected sets (4, 12, 16, 20, 28, and 32) as controls for every six sets (1–6, 7–12, 13–18, 19–24, 25–30, 31–32). The two control checks had no significant difference in phenotypes of interest between sets (Supplementary Figure S1).

14-day-old seedlings were transferred for 7 days to a 10 °C growth chamber with identical light-dark cycles. Watering was done once every other day to account for water depletion in boxes and to buffer temperature fluctuations. At the end of the treatment, the middle segment of each seedling was collected for the electrolyte leakage assay; other seedlings’ shoot tissues were collected for catalase and anthocyanin activity assays.

The remaining stressed seedlings were moved back to regular growth conditions mentioned above for 7 days, with similar watering schedule of once every other day. Low-temperature seedling survivability was recorded after 7 days of recovery.

### 2.3. Measurement of Electrolyte Leakage (EL)

EL measurements were done as previous described, with slight modifications [11,40]. Briefly, at the end of the 7-day stress period, the middle region of the second leaf was collected from three individual seedlings and cut into four equally sized segments. The pieces were washed in distilled water (conductivity ≤2 µS/cm), transferred into screw-cap glass tubes filled with 5 mL of distilled water, and shaken at 200 rpm for 60 min at room temperature to release cellular electrolytes from damaged tissues. EL of each of the three replicate samples was measured by taking out 120 µL of the solution and adding it into the well of a hand-held LAQUAtwin B-771 conductivity meter (Horiba Scientific, Japan). After the initial reading, leaf samples were boiled for 20 min to release the total cellular electrolytes. Samples were cooled to room temperature, shaken again at 200 rpm for 30 min, and the final electrolyte leakage reading was done. EL percentage was determined as [(initial EL)/(total EL)] × 100.

### 2.4. Measurement of Catalase Activity (CAT)

CAT assays were done as previously described, with slight modifications [11]. Briefly, proteins from 100 mg of finely ground cold stressed tissues (mixture of at least three plants) were extracted with 700 µL of phosphate buffer (pH 7.0). Samples underwent a centrifugation at 14,000× *g* for 5 min, and the supernatant was collected and diluted 1:1 with the same phosphate buffer. Total protein concentration was measured using the Bradford reagent (Fisher Scientific, Hampton, NH, USA). For the CAT activity assay, filter paper discs (8 mm diameter) were soaked for 5 sec in 5-fold diluted protein extracts, placed on a paper towel for another 5 sec to get rid of excessive extracts. Discs were then immediately placed at the bottom of a 50-mL glass beaker containing 30 mL of freshly made 1% H_2_O_2_. CAT activity was determined as the time the disc took to reach the surface of the H_2_O_2_ solution (30 mm in height). CAT activity was calculated as Activity Units = [(30 mm/time in sec)/(total protein concentration in mg)].

### 2.5. Measurement of Anthocyanin Activity (ANT)

ANT assays were done as previously described, with slight modifications [11]. Briefly, phenolic compounds were isolated from 100 mg of finely ground cold stressed and control tissues (mixture of at least three plants) with 700 µL of 70:30:1 methanol:water:trifluoroacetic acid extraction buffer. Samples were incubated in the dark overnight at room temperature. Samples were collected after one round of centrifugation at 14,000× *g* for 20 min. The supernatant was collected and filtered through a 13-mm syringe containing one layer of 0.2-µm PTFE membrane. The total phenolic compound content in mg was calculated as {[(OD_520_ − OD_700_) × 449.2 × 103] × (1/26900)}. 25 µL of this extract were mixed with 200 mL of 0.25 mM 2,2-diphenyl-1-picrylhydrazyl (Sigma). The mixture was incubated with shaking at 200 rpm for 30 min. Absorbance at 517 mm was recorded. Anthocyanin activity was calculated as ANT activity = {[(OD_517_ of blank − OD_517_ of sample)/OD_517_ of blank] × 100/(total phenolic compound content in mg)}.

### 2.6. Measurement of Low Temperature Seedling Survivability (LTSS)

Low temperature seedling survivability was determined after 7 days of recovery via visual inspection, as described previously [11,37,40]. Specifically, alive seedlings were mostly green while dead seedlings were mostly wilted and/or bleached in the meristem region. The mean percent survivability was calculated as [(number of alive seedlings)/(number of stressed plants) × 100].

### 2.7. Genome-Wide Association Study (GWAS)-Based Quantitative Trait Locus (QTL) Mapping

The mean values of three trials from each individual phenotyping assay were averaged, and that average was used for GWAS-Based QTL mapping via the publicly available Cornell GWAS mapping pipeline [38] as described previously [40]. Briefly, this pipeline runs in conjunction with 700,000 SNPs of the High-Density Rice Array (HDRA) [38,44,45] to generate Manhattan and quantile-quantile (QQ) plots. Reference SNPs for HDRA were from the *temperate japonica* cultivar Nipponbare and the *indica* cultivar 93-11 representing the *JAPONICA* and *INDICA* subspecies, respectively [45]. The pipeline uses a variance component approach implemented in the publicly available software, efficient (linear) mixed-model association eXpedited (EMMAX), to map HDRA-derived 700 K SNPs to phenotype and to account for population structure by calculating and including a kinship matrix as a covariate, which helps to distinguish true associations from false positives due to relatedness between accessions. The kinship matrix was calculated using identity by state (IBS) to group identical allele states while excluding shared ancestry. Default settings for the Cornell GWAS mapping pipeline were set as minor allele frequency (MAF) >10%, minor allele count (MAC) >1, percent missing allele <30%, false discovery rate = 0.1, and best SNP limit = 0.05.

### 2.8. QTL Mapping

Manhattan plots from GWAS runs were extracted as Excel files and –log10(*p*-value) values were calculated for each SNP. QTL were assigned to genomic regions with at least 3 significant SNPs within a 1 Mb segment. Significant SNPs had the –log10(*expect*) value of 3.0, corresponding to –log10(*p*-value) of 4.3, 3.6, and 2.8 for LTSS, EL, and CAT and ANT activities, respectively, based on generated QQ plots (Supplementary Figure S2).

### 2.9. Gene Identification and Gene Ontology (GO) Term Analysis

Genes within selected QTL were found via Allele Finder with the following settings [38]: “Dataset” = “HDR6.4_RDP1-RDP2-NIAS-sativa-only”, and “Rice sub populations for query” = “admixed”, “admixed-indica”, “admixed-japonica”, “aromatic”, “aus”, “indica”, “temperate-japonica”, “tropical-japonica”. GO terms for these genes were retrieved via PhytoMine with gene names in MSU format and “Oryza sativa v7.0” selected as the the “Organism”. Table GO term enrichment was done with the GO Term Enrichment tool from PlantRegMap, with “Oryza sativa subsp. japonica” selected for “Species”, and “≤ 0.05” for “*p*-value”. GO term clustering and visualization were done via MonaGO (https://monago.erc.monash.edu/, 21 August 2021) with GO IDs and their associated *p*-values and “number of overlapping genes” selected for the “Distance Measurement”. All genes used in this analysis were present in Nipponbare (*temperate japonica*) and 93-11 (*indica*), both of which were used as reference genomes for the High-Density Rice Array [45].

### 2.10. Correlation Analysis and Statistical Analysis

Correlation analyses between phenotypes were done using the OriginLab Pro 2018 SR1 b9.5.1.195 program (OriginLab Corp., Northampton, MA, USA). Linear, Boltzmann, and exponential decay correlations were calculated, and best-fitting options selected. Significant differences for all cold tolerance phenotypes between the three clusters were calculated using OriginLab Pro 2018 SR1 b9.5.1.195 with unpaired Student’s *t*-test option selected with an addition of Welch’s test selected as necessary since the populations between the three clusters were not equal and had to be calculated accordingly.

## 3. Results

### 3.1. Summary of Low Temperature Seedling Survivability (LTSS) Results

**Figure 2:**
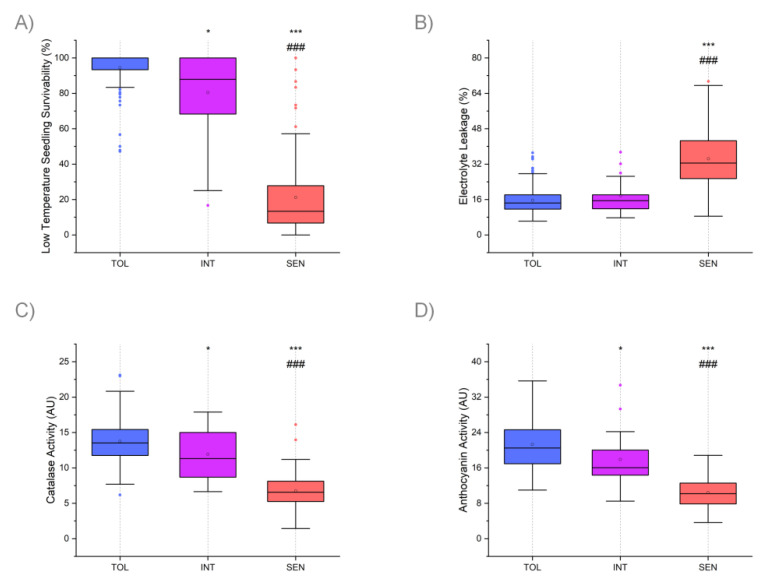
Box plots of mean values for four phenotypes after a 7-day-10 °C chilling treatment for accessions of three cold tolerance clusters (see Figure 1 for details). (**A**) Low Temperature Seedling Survivability (LTSS) values for rice accessions of the three cold tolerance clusters. (**B**) Electrolyte Leakage (EL) values measuring membrane integrity after chilling stress for rice accessions of the three cold tolerance clusters. (**C**) Catalase (CAT) Activity values measuring enzymatic antioxidative activity for rice accessions of the three cold tolerance clusters. (**D**) Anthocyanin (ANT) Activity values measuring non-enzymatic antioxidative activity for rice accessions of the three cold tolerance clusters. TOL: Cold Tolerant; INT: Intermediate Cold Tolerant; SEN: Cold Sensitive. Significant differences compared to the TOL cluster are labeled by the symbol “*”. Significant differences compared to the INT cluster are labeled with the symbol “#”. ***/###: *p* < 0.001; *: *p* < 0.05.

To correlate enzymatic and non-enzymatic antioxidative activities during chilling stress with general seedling survivability, two-week old seedlings were exposed for one week to continuous chilling stress of 10 °C. After one week of recovery at regular growth condition, seedlings that were green and healthy were counted as successfully recovered while those that were white and/or wilted were counted as dead. For statistical analysis, Rice Diversity Panel 1 (RDP1) accessions were assigned to three clusters based on their genetic background (see Materials and Methods). This classification was further supported by a high degree of consistency between LTSS (and electrolyte leakage) results reported by Shimoyama et al. 2020 [40] and results shown here using the same RDP1 accessions (Supplementary Figure S3). The mean LTSS score for the Tolerant cluster was 94.5%; for the Intermediate cluster, 80.4%; and for the Sensitive cluster, 21.2% (Table 1; Figure 2A). Compared to the Tolerant cluster, LTSS scores for the Intermediate and Sensitive clusters were significantly lower, with *p*-values (Student’s *t*-test) of 1.53 × 10^−02^ and 3.52 × 10^−72^, respectively. Compared to the Intermediate cluster, LTSS scores for the Sensitive cluster were significantly lower, with a *p*-value (Student’s *t*-test) of 4.04 × 10^−11^. The variances were similar and consistent between the three experiments (Supplementary Table S1). Since the Tolerant cluster made up approx. 60% of the rice collection used in this study, we expected LTSS scores for the population to be skewed toward the high end. As shown in Supplementary Figure S4a, 41 accessions had LTSS scores in the range of 80–90%, and 187 accessions had LTSS scores in the range of 90–100%, while the mean LTSS score for all 370 accessions was 67.3%. Thus, as expected, 61.6% of the accessions had LTSS scores at the higher end, between 80–100%. All three experiments produced a similar distribution profile (Supplementary Figure S5A–C).

### 3.2. Summary of Electrolyte Leakage (EL) Results

To correlate enzymatic and non-enzymatic antioxidative activities during chilling stress with membrane damage sustained during cold temperature exposure, EL in leaves was determined immediately after the 7-day-10 °C chilling treatment. For consistency, the middle portion of the second leaf of young seedlings was used for the EL measurements. The mean percent EL for the Tolerant cluster was 15.7%; for the Intermediate cluster, 17.7%; and for the Sensitive cluster, 34.4% (Table 1; Figure 2B). Compared to the Tolerant cluster, EL scores for the Sensitive clusters were significantly higher, with *p*-values (Student’s *t*-test) of 1.39 × 10^−34^, respectively. Compared to the Intermediate cluster, EL scores for the Sensitive cluster were significantly higher, with a *p*-value (Student’s *t*-test) of 1.81 × 10^−10^. The variances were similar and consistent between the three experiments (Supplementary Table S1). Since the Tolerant cluster made up approx. 60% of the rice collection used in this study, we expected EL scores for the population to be skewed toward the low end. As shown in Supplementary Figure S4b, 131 accessions had EL scores in the range of 5–15%, and 113 accessions had EL scores in the range of 15–25%, while the mean EL score for all 370 accessions was 22.5%. All three experiments produced a similar distribution profile (Supplementary Figure S5D–F).

### 3.3. Summary of Catalase (CAT) Activity Results

Immediately after the 7-day-10 °C chilling treatment, whole seedling tissues, including shoots and roots, were collected to determine the antioxidative activity of CAT, a member of the enzymatic pathway for turning over reactive oxygen species (ROS). The mean CAT Activity Units (AU) for the Tolerant cluster was 13.8 AU; for the Intermediate cluster, 11.9 AU; and for the Sensitive cluster, 6.8 AU (Table 1; Figure 2C). The variances were similar and consistent between the three experiments (Supplementary Table S1). Compared to the Tolerant cluster, CAT AUs for the Intermediate and Sensitive clusters were significantly lower, with *p*-values (Student’s *t*-test) of 2.05 × 10^−02^ and 1.31 × 10^−71^, respectively. Compared to the Intermediate cluster, CAT AUs for the Sensitive cluster were significantly lower, with a *p*-value (Student’s *t*-test) of 2.74 × 10^−07^. As shown in Supplementary Figure S4c and Supplementary Figure S6a–c, compared to LTSS and EL, CAT activity scores for the 370 RDP1 accessions had a normal distribution. CAT activities for 70 accessions representing the three tolerance clusters were similar between the three clusters under warm control conditions (Supplementary Figure S7a).

### 3.4. Summary of Anthocyanin (ANT) Activity Results

Immediately after the 7-day-10 °C chilling treatment, whole seedling tissues, including shoots and roots, were collected to determine the antioxidative activity of ANTs, members of the non-enzymatic pathway for turning over ROS. The mean ANT Activity Units for the Tolerant cluster was 21.3 AU; for the Intermediate cluster, 17.9 AU; and for the Sensitive cluster, 10.4 AU (Table 1; Figure 2D). The variances were similar and consistent between the three experiments (Supplementary Table S1). Compared to the Tolerant cluster, ANT AUs for the Intermediate and Sensitive clusters were significantly lower, with *p*-values (Student’s *t*-test) of 1.74 × 10^−02^ and 2.62 × 10^−74^, respectively. Compared to the Intermediate cluster, ANT AUs for the Sensitive cluster were significantly lower, with a *p*-value (Student’s *t*-test) of 7.99 × 10^−06^. As shown in Supplementary Figure S4d and Supplementary Figure S6d–f, compared to LTSS and EL, ANT activity scores for the 370 RDP1 accessions had a normal distribution. ANT activities for 70 accessions representing the three tolerance clusters were similar between the three clusters under warm control conditions (Supplementary Figure S7b).

### 3.5. Correlations between LTSS, EL, CAT Activity, and ANT Activity after Low Temperature Treatment

#### 3.5.1. Correlation between EL and LTSS

**Figure 3:**
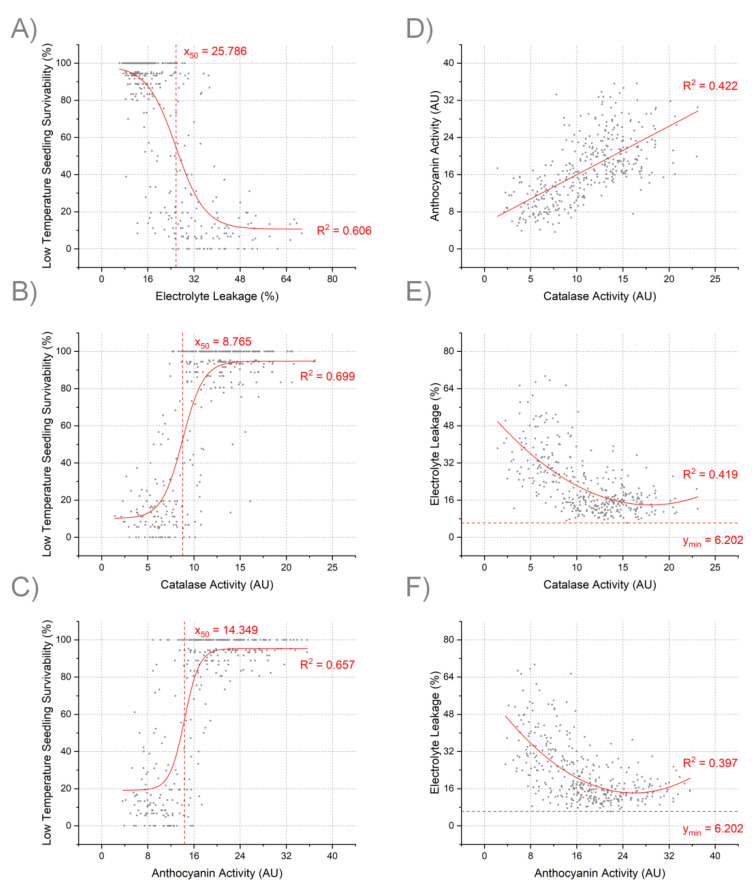
Correlation analyses of four chilling tolerance phenotypes after a 7-day-10 °C treatment. Each dot shows the correlation for one accession. (**A**–**C**) Correlations between LTSS and EL, CAT, and ANT, respectively. A Boltzmann Sigmoid fit is shown based on best-fit model calculations. X_50_ and the line y = X_50_ show values at which EL, CAT, ANT are halfway between high LTSS and low LTSS. (**D**) Correlations between CAT and ANT activities. A linear fit is shown based on best-fit model calculations. (**E**,**F**) Correlations between EL, and CAT and ANT, respectively. An Exponential Decay fit is shown based on best-fit model calculations. The line x = y_min_ shows the lowest EL value after reaching the plateau for the curve.

As LTSS scores were determined after a 7-day recovery period while EL measurements were done immediately after the chilling treatment, it was important to investigate how well EL and LTSS correlate to determine whether EL could be used to predict how well rice plant can recover after the 10 °C chilling stress treatment. Interestingly, there was a non-linear Boltzmann type negative correlation between the two phenotypes (*R^2^* = 0.606) (Figure 3A). The Boltzmann sigmoidal curve determined the x-value responsible for the 50% LTSS threshold to be 25.8% EL. There were 211 accessions (57%) with EL scores below this threshold that achieved at least 80% seedling survivability.

#### 3.5.2. Correlation between CAT Activity and LTSS

As CAT activity assays were done at the end of the cold exposure and LTSS scores determined one week later, a correlation analysis between CAT activity and LTSS was done to determine how well catalase activity could predict seedling survivability. There was a Boltzmann type positive correlation between CAT activity immediately after the 10 °C chilling stress and the ability of seedlings to recover one week later (*R^2^* = 0.699) (Figure 3B). The Boltzmann sigmoidal curve determined the x-value responsible for the 50% LTSS threshold to be 8.77 AU of CAT activity. There were 219 accessions (59%) with CAT activities above the 8.77 AU threshold that successfully achieved at least 80% seedling survivability.

#### 3.5.3. Correlation between ANT Activity and LTSS

Similarly, as ANT activity assays were done at the end of the cold exposure and LTSS scores determined one week later, a correlation analysis between ANT activity and LTSS scores was done to determine how well anthocyanin activity could predict seedling survivability. This showed that, similar to the CAT activity-LTSS correlation, there was a Boltzmann type positive correlation between ANT activity immediately after the 10 °C chilling stress and the ability of seedlings to recover one week later (*R^2^* = 0.657) (Figure 3C). The Boltzmann sigmoidal curve determined the x-value responsible for the 50% LTSS threshold to be 14.35 AU of ANT activity. There were 209 accessions (56.5%) with anthocyanin activities above the 14.35 AU threshold that successfully achieved at least 80% seedling survivability.

#### 3.5.4. Correlation between EL and CAT Activity

As both CAT activity assays and EL measurements were done at the end of the cold exposure, a correlation analysis between CAT activity and EL was done to determine how well catalase antioxidative activity could predict the degree of membrane damage sustained during the cold stress period. There was an exponential decay correlation between CAT activity and cell membrane damage immediately after the 10 °C chilling stress (*R^2^* = 0.419; Figure 3E), that is, the higher CAT AUs, the lower EL scores. The low EL plateau (y-minimum line in Figure 3E) reached by most RDP1 accessions with the highest CAT AUs was at 6.6%, which is similar to the baseline EL level of undamaged rice leaves grown under warm control conditions.

#### 3.5.5. Correlation between ANT Activity and EL

Likewise, both ANT activity assays and EL measurements were done at the end of the cold exposure, a correlation analysis between ANT activity and EL was done to determine how well non-enzymatic anthocyanin antioxidative activity could predict the degree of membrane damage sustained during the cold stress period. This showed that, similar to the CAT activity-EL correlation, there was an exponential decay correlation between ANT activity and plasma membrane damage sustained immediately after the 10 °C chilling stress period (*R^2^* = 0.397) (Figure 3F). Again, the baseline low EL plateau (y-minimum line in Figure 3F) reached by most RDP1 accessions with the highest ANT AUs was at 6.6%.

#### 3.5.6. Correlation between CAT Activity and ANT Activity

As CAT and ANT activity assays were done to measure seedling’s antioxidative ability, a correlation analysis between CAT activity and ANT activity was done to determine whether enzymatic and non-enzymatic pathways were aligned to cope with cold temperature stress. The analysis showed that there was a positive linear correlation between CAT and ANT activity immediately after the 10 °C chilling stress (*R^2^* = 0.422) (Figure 3D).

### 3.6. Genome-Wide Association Study (GWAS)-Based Mapping of Four Phenotypes

**Figure 4:**
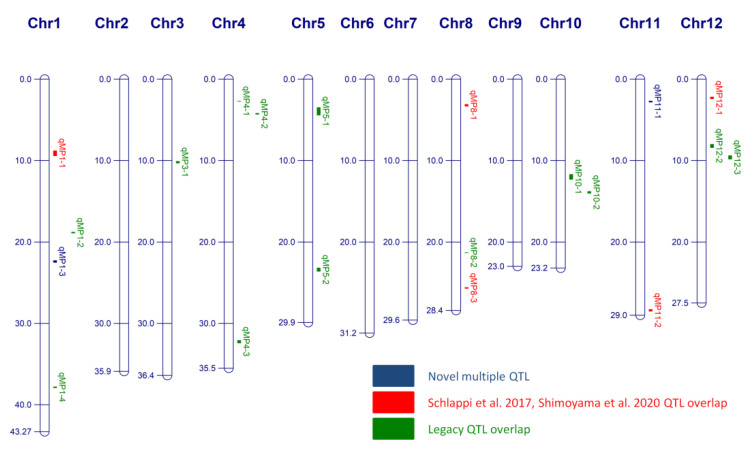
Genomic locations of 20 *Multiple-Phenotype* (*MP*) QTL. Numbers along the chromosomes (Chr) are million-base-pair (Mbp) units. Boxes show regions containing at least one antioxidant QTL (*qCAT*; *qANT*) overlapping with another QTL (*qLTSS*; *qEL*; *qCAT*; or *qANT*). Blue boxes show regions of novel cold stress tolerance QTL identified in this study; red boxes show regions of *qMPs* overlapping with cold stress tolerance QTL reported in Schläppi et al. 2017 [37] and Shimoyama et al. 2020 [40]; green boxes show *qMPs* overlapping with some legacy seedling stage cold stress tolerance QTL reported in the last five years (Lv et al. 2016 [46], and Wang et al. 2016 [47]).

To identify single nucleotide polymorphisms (SNPs) associated with the four phenotypes LTSS, EL, CAT activity, and ANT activity, phenotypic data for the 370 RDP1 accessions were run through the Cornell GWAS mapping pipeline [38]. At the –log10(expect) value of 3.0, the cutoff *p*-value for significant SNPs for the four phenotypes were determined to be 4.3 for LTSS, 3.6 for EL, and 2.8 for both CAT activity and ANT activity (Supplementary Figure S2). Quantitative trait loci (QTL) were identified as a region containing ≥3 SNPs within a one-million-base-pair region. This yielded 25 individual LTSS, 30 EL, 31 CAT activity, and 34 ANT activity QTL (Supplementary Table S2). Of these, 22 QTL overlapped with two phenotypes and 3 QTL overlapped with three phenotypes, and 20 QTL overlapped with at least one of the antioxidant activity QTL. Since the major goal of this study was to assess the contribution of antioxidant activity to membrane integrity and seedling survivability during chilling stress, we assigned *Multiple-Phenotype* (*MP*) QTL to regions that had at least one antioxidant activity QTL overlapping with other cold stress response QTL (Table 2; Figure 4). The 20 *qMP* overlapping with at least one antioxidant activity QTL are shown in Figure 4 while a list of all individual QTL is shown in Supplementary Table S2. The most significant *MP* QTL was *qMP10-2* at 13.8–14.0 Mb on chromosome 10, with a LOD score of 6.80 (Table 2).

### 3.7. GO Term Analyses of Annotated Genes within Multiple-Phenotype QTL

**Figure 5:**
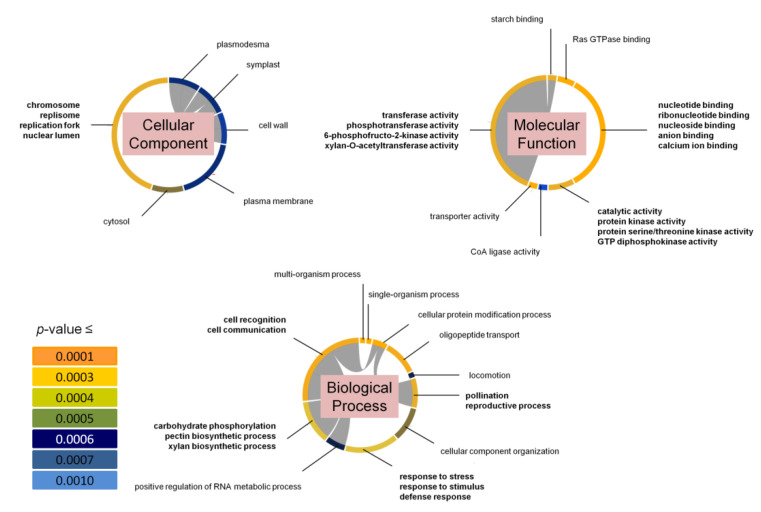
Gene Ontology (GO) term enrichment of annotated genes found in regions of the 20 *Multiple-Phenotype* QTL shown in Figure 4. The segment color corresponds to different *p* values (as shown). The segment length corresponds to the number of genes contributing to that GO term/cluster. In Bold: multiple GO terms clustered together based on overlapping genes.

The chromosomal regions spanning those 20 *MP* QTL contained 579 annotated genes. However, 161 of them were annotated as either transposons, retrotransposons, expressed proteins, or hypothetical protein coding genes. These genes were not used for GO term analysis and enrichment, leaving 418 genes for these analyses. For the GO term analysis, there were 94 terms for the Biological Process run, 56 terms for the Molecular Function run, and 14 terms for the Cellular Component run with *p* values ≤ 0.05. These GO terms were further enriched using a *p* value cutoff of 0.001. This cutoff enriched 20 GO terms for Biological Process; 19 GO terms for Molecular Function; and nine GO terms for Cellular Component (Figure 5).

For Biological Process, 17 GO terms were selected after accounting for similarities. From these, there were four distinct clusters shared by at least half of the contributing genes. The first cluster was for “cell recognition” and “cell communication”. The second cluster was for “pollination” and “reproductive process”. The third cluster was for “response to stress”, “response to stimulus”, and “defense response”. And the fourth cluster was “carbohydrate phosphorylation”, “pectin biosynthetic process”, and “xylan biosynthetic process”. Within the first cluster, the terms “cellular protein modification process” and “oligo peptide transport” were the most significant (Figure 5).

For Molecular Function, 17 GO terms were selected after accounting for similarities. From these, there were three distinct clusters shared by at least half of the contributing genes. The first cluster was for “nucleotide binding”, “ribonucleotide binding”, “nucleoside binding”, “anion binding”, and “calcium ion binding”. The second cluster was for “catalytic activity”, “protein kinase activity”, “protein serine/threonine kinase activity”, and “GTP diphosphokinase activity”. And the third cluster was for “transferase activity”, “phosphotransferase activity”, “6-phosphofructose-2-kinase activity”, “xylan-O-acetyltransferase activity”. Within the first cluster, the terms “Ras GTPase binding” and “transporter activity” were the most significant (Figure 5).

For Cellular Component GO terms, of the nine enriched GO terms, one distinct cluster was shared by at least half of the contributing genes. This cluster was for “chromosome”, “replisome”, “replication fork”, and “nuclear lumen”. Within this cluster, the terms “cytosol” and “plasmodesma” were the most significant (Figure 5).

## 4. Discussion

### 4.1. Cold Stress Tolerance Scores of Rice Accessions Cluster with Their Respective Subgroups

The purpose of this study was to correlate four cold stress tolerance responses of 370 Rice Diversity 1 (RDP1) accessions with each other to further our understanding of biochemical and genetic response mechanisms involved in establishing cold tolerance in rice. We moreover wanted to identify robust parameters immediately after the cold temperature stress exposure to predict the overall low-temperature seedling survivability (LTSS) after one week of recovery. To this end, we assessed at the end of the cold stress period percent electrolyte leakage (EL) as a measure of the degree of membrane damage sustained during that period, and catalase (CAT) and anthocyanin (ANT) activities as a measure of enzymatic and non-enzymatic antioxidant activities, respectively, to detoxify reactive oxygen species (ROS) produced during the cold stress period. A distribution analysis of the scores for all four low-temperature stress phenotypes indicated that the 370 RDP1 accessions generally cluster with the genetic backgrounds of their subgroups. This means that accessions from the *temperate japonica* and *tropical japonica* subgroups, and admixes between the two subgroups, classified as the *JAPONICA* subspecies of rice, can be assigned to the cold Tolerant cluster, because they generally had low EL (i.e., low membrane damage), high CAT and ANT (i.e., high ROS detoxification) activities, and overall high LTSS (Table 1; Figure 2). In contrast, accessions from the *aus* and *indica* subgroups, and admixes between the two subgroups, classified as the *INDICA* subspecies of rice, can be assigned to the cold Sensitive cluster, because they generally had high EL (i.e., high membrane damage), low CAT and ANT (i.e., low ROS detoxification) activities, and overall low LTSS (Table 1; Figure 2). Accessions from the *aromatic* subgroup and admixes between subgroups of the *INDICA* and *JAPONICA* subspecies can be assigned to the Intermediate cold tolerant cluster, because the means of their phenotypic scores ranged between those of the Tolerant and the Sensitive clusters (Table 1; Figure 2). This finding is consistent with previous publications showing that accessions from the *JAPONICA* subspecies are generally more-cold tolerant than accessions from the *INDICA* subspecies, which is a good prerequisite for mapping genomic regions containing genes involved in cold tolerance responses [11,40].

Although previous publications suggested a linear correlation between EL and LTSS after cold stress exposure [40,48], our results suggest a sigmoidal relationship between those two parameters (Figure 3). Under conditions tested here, there was a threshold value of 25.8% EL below which LTSS scores were high in a relative non-linear fashion and above which LTSS scores were low. However, as previously reported [40,48], some rice accessions had low EL and low LTSS or high EL and high LTSS scores, suggesting that besides membrane damage, other molecular and physiological events determine seedling survivability during the one-week recovery period. Consistent with our EL data, correlations between CAT and ANT antioxidant activities and LTSS also followed a sigmoid relationship (Figure 3). Above the thresholds of 8.8 AU for CAT and 14.4 AU for ANT, LTSS scores were high and did not increase much at higher AU values while below those values, LTSS scores were low and did not decrease much at lower AU values. Interestingly, a distribution analysis of percent EL and LTSS showed that the values were skewed toward one end or another, while antioxidative activities followed a normal distribution (Supplementary Figure S4).

It is worth noting that within the Tolerant cluster, most of the *temperate japonica* accessions had high antioxidative activity and low membrane damage, while within the Sensitive cluster, most *aus* accessions had low antioxidative activity and high membrane damage. Meanwhile, within these two clusters, accessions from the *tropical japonica* and *indica* subgroups had a wider distribution of their antioxidative activity and membrane damage scores. One explanation for this is that because low-temperature tolerance in plants is a complex trait [8,11,37,40], every molecular/genetic pathway involved needs to be fine-tuned to perfection [8,9,10,49,50]. As low temperatures negatively affect plants in multiple ways, including nutrient uptake, resources within plants might become scarce and trade-offs need to be made [9,10]. Therefore, it might be too “expensive” for some plants to keep increasing antioxidative activities to fully reduce ROS to pre-stress levels. Our results showed that some rice accessions with high LTSS values had antioxidative activity scores barely above the measured threshold levels. Likewise, some accessions had EL scores close to the threshold level yet were still able to successfully recover with high LTSS values. This would explain observations showing that in *indica* accessions, some defense and abiotic stress tolerance genes are upregulated only when intracellular ROS abundance reaches a certain level [51]. It is possible that similarly, certain cold tolerance genes are only upregulated when the cell membrane suffers an adequate degree of damage. This would explain why many tropical *japonica* and some *indica* accessions with good LTSS scores can tolerate relatively high membrane damage and low antioxidant activity levels, because these conditions might activate stress response genes fixing damaged membranes during recovery growth. Meanwhile, *temperate japonica* accessions with low membrane damage can afford high antioxidative activities to detoxify ROS and achieve high LTSS. Additionally, multiple transcriptomic studies have shown that cold stress responsive genes are regulated differently depending on the stress intensity [52,53,54,55,56]. Taken together, different rice accessions might balance different cold stress tolerance response strategies with the requirement for growth and development during the recovery period.

### 4.2. Genome-Wide Association Study (GWAS)-Based Mapping of Four Cold Tolerance Traits

Based on our previous experience with the GWAS pipeline used in this study, we are confident that the 20 QTL associated with two or three overlapping cold tolerance traits are robust indicators not only for genes regulating membrane integrity and overall seedling survival after cold stress exposure in rice, but also for genes that control antioxidative activity to counteract ROS production during stress exposure. Of these 20 QTL, 18 overlapped with previously mapped QTL associated with various cold tolerance traits (Figure 4), while 10 overlapped with previously mapped multiple-trait QTL using a similar subset of 354 RDP1 accessions [40]. 14 QTL overlapped with previous mapping studies using different rice populations [37,40,46,47] (Table 2), whereas the two QTL *qMP1-3* and *qMP11-1* at 22 Mb on chromosome 1 and 2 Mb on chromosome 11, respectively, appear to be novel cold tolerance QTL. Because the 20 QTL found in this study span regions of less than 1 Mb, they will help narrow down cold tolerance candidate genes of previously mapped QTL to smaller segments.

Several genes within some of the 20 QTL were previously shown to contribute to cold tolerance in rice [57,58]. For instance, upregulation of *OsCTB4A* located within *qMP4-1* increased ATP synthase activity and ATP content, enhanced seed setting, and improved yield under cold stress conditions [59]. *OsFBK15*, one of the major factors affecting cold tolerance in rice at the booting stage, is closely linked to *qMP4-3* [60,61]. *OsRH42* is found within *qMP8-1* and was previously shown to be antagonistic for pre-mRNA splicing in plant under chilling stress [62]. The MAP kinase encoding gene *OsMPK5* is found within *qMP3-1* and was shown to be one of the regulators of gene expression and antioxidants under different stress conditions, including drought, cold and high salinity [63,64,65,66]. The MYB transcription factor encoding gene *OsMYB3R-2* was found to enhance low temperature tolerance via alteration of the cell cycle and is closely linked to *qMP1-4* [67,68,69]. Overexpression of *OsLAC13* was previously shown to induce hydrogen peroxide production and mitochondrial damage [70] and is linked to *qMP5-2*, a QTL associated with EL and CAT activity traits. The gibberellins-2-oxidase encoding gene *OsGA2OX1* is within *qMP5-1* and was previously shown to coordinate chilling tolerance and growth in rice [71].

Interestingly, none of the multiple or single phenotype QTL we identified mapped to any of the four catalase genes in rice, which are found at 0.8 Mb on chromosome (chr.) 2 (*OsCATA*), at 30.9 Mb on chr. 6 (*OsCATB*), at 1.7 Mb on chr. 3 (*OsCATC*), and at 23.7 Mb on chr. 4 (*OsCATD*) [21], despite the fact that CAT activity was one of the phenotypes used for mapping. This can be explained with a lack of polymorphism associated with CAT genes, or with significant polymorphism associated with genes regulating CAT activity. However, there are three peroxidase genes associated with *qMP1-1*, *LOC_Os01g15810*, *LOC_Os01g15830*, *LOC_Os01g16450*, and one gene, *LOC_Os05g06970*, with *qMP5-1*. The gene *LOC_Os01g15830* is also known as *OsPOX1* and has been shown to be a cold-responsive gene in rice [72]. As catalases are members of the broader peroxidase family [20], it is reasonable that CAT activity QTL can be associated with peroxidase genes. Additionally, since QTL mapping was also done using the ANT activity trait, four anthocyanin reductase genes, *LOC_Os04g53830*, *LOC_Os04g53850*, *LOC_Os04g53860*, and *LOC_Os04g53920* were found to be associated with *qMP4-3*, and the 1 flavone-methyltransferase gene, *LOC_Os08g06100*, with *qMP8-1*. As flavones, anthocyanins, and its sugar-free counterpart, anthocyanidins, work together in the non-enzymatic pathway of ROS turnover [25], it is reasonable that these genes are associated with ANT and/or CAT QTL.

### 4.3. Gene Ontology (GO) Term Analysis of Genes Found in 20 Multiple-Phenotype QTL

The GO term enrichment and clustering data of genes within multi-phenotype QTL revealed three major pathways that can influence the plant’s antioxidative activity, and its overall survivability during and after cold stress exposure.

The first pathway appears to involve signal transduction between cells, because the cluster “cell recognition”/“cell communication”, and the two GO terms “plasmodesma” and “symplast” were filtered out (Figure 5). Because plants are sessile and need to adapt to environmental challenges, complex signaling cascades need to be adjusted accordingly [8,11,49]. Studies have shown that for the plant to utilize ROS as signaling agents, CAT activity is often dowregulated early during stress exposure [73,74]. However, as the stress prolongs, ROS must be turned over due to their destructive nature towards cellular components [14,15,16]. Hence, it makes sense that from mapping cold tolerance candidate genes via antioxidative activity, a complex network for signaling between cells is revealed.

The second pathway appears to involve maintenance of the plasma membrane and cell wall integrity, as the cluster “carbohydrate phosphorylation”/“pectin biosynthetic process”/“xylan biosynthetic process”, and the two GO terms, “cell wall” and “plasma membrane” were filtered out (Figure 5). Since the lipid composition of the plasma membrane is sensitive to temperature fluctuations [10,13], not being able to maintain the plasma membrane’s integrity poses a risk for membrane lesions, which can lead to cell death [11,13]. Meanwhile, pectin and xylan are two components of the plant cell wall [75]. It has been shown that cell wall softening is crucial for cell elongation and growth, while cell wall stiffening plays an important role in both abiotic and biotic stress tolerance [76,77,78]. Furthermore, modifications of the cell wall may trigger other downstream signaling events to activate defense pathways [79]. Filtering out this pathway from GO enrichment makes sense as electrolyte leakage, which measures the degree of membrane damage, was used as a parameter for mapping.

The third pathway appears to address dynamic molecular events involving DNA and RNA, as the cluster “chromosome”/“replisome”/“replication fork”/“nuclear lumen”, and the cluster “nucleotide binding”/“ribonucleotide binding”/“nucleoside binding”/“anion binding”/“calcium ion binding” was filtered out (Figure 5). These clusters suggest that regulation of DNA replication and possibly chromatin structure play a role in cold stress tolerance responses in rice. Nucleosomes contain “nucleotide binding” proteins and their structure determines accessibility of DNA sequences in transcription, DNA replication, repair, and recombination [80,81,82]. Therefore, cold temperature mediated nucleosome remodeling might help establishing gene expression states involved in cold stress tolerance responses, while the term “ribonucleotide binding” suggests contributions of ATP-binding kinases and GTP-binding G-proteins in this process [83].

Taken together, the GO term enrichment helps further our understanding of how ROS and antioxidative activity contribute to maintaining membrane integrity and general survivability during cold stress exposure by playing roles in complex signaling networks, plasma membrane and cell wall architecture, and DNA replication, chromatin remodeling, and regulation of gene expression.

## 5. Conclusions

This study provides a novel and fine-tuned list of four types of cold tolerance trait QTL and associated candidate genes and genetic pathways in an important crop plant using 370 RPD1 *O. sativa* accessions and a GWAS-based mapping pipeline. We show that at the end of the chilling stress treatment, different levels of either enzymatic or non-enzymatic antioxidative activity predict the survivability potential of rice plants after a one-week recovery period. Rice breeders could use this ability of cold stressed rice plants to turn over ROS as a proxy to predict cold stress tolerance outcomes of their breeding lines. Interestingly, even with antioxidative activity as another filtering layer to investigate cold stress tolerance response mechanisms, numerous previously identified QTL regions were uncovered again. This indicates a close association between antioxidative activity and physiological cold stress tolerance response mechanisms. Within the QTL regions, an enrichment of genes belonging to the three pathways of signal transduction, maintenance of the plasma membrane and cell wall integrity, and nucleic acids metabolism, was identified at a high significance level. The high degree of overlap between antioxidant activity QTL and membrane integrity and overall seedling survivability QTL shows that an appropriate regulation of ROS homeostasis during cold stress exposure is critical for rice cold tolerance at the young seedling stage. A detailed investigation of candidate genes belonging to the three pathways, from SNP allele frequency analysis to transcriptomic studies, will help to improve cold tolerance potentials not only of Asian rice, but most likely, also of other important crop plants.

## Figures and Tables

**Table 1:** Means and standard error for four phenotyping assays using 370 RDP1 *O. sativa* accessions after a 7-day-10 °C chilling treatment. ARO: *aromatic*; AUS: *aus*; IND: *indica*; MIX-I: admixed accessions within *INDICA*; MIX-J: admixed accessions within *JAPONICA*; TEJ: *temperate japonica*; TRJ: *tropical japonica*; TOL: Cold Tolerant; INT: Intermediate Cold Tolerant; SEN: Cold Sensitive.

Genetic Background	Number of Accessions	Low Temperature Seedling Survivability (%)	Electrolyte Leakage (%)	Catalase Activity (AU)	Anthocyanin Activity (AU)
*ADMIX*	11	80.22 ± 2.05	15.74 ± 2.02	11.89 ± 1.16	15.33 ± 1.14
*ARO*	11	80.60 ± 3.32	19.65 ± 2.41	11.96 ± 1.00	20.47 ± 1.45
*AUS*	53	13.11 ± 1.76	36.43 ± 1.70	6.68 ± 0.26	10.30 ± 0.44
*IND*	74	27.44 ± 1.46	32.47 ± 1.22	6.77 ± 0.27	10.59 ± 0.45
*MIX-I*	6	14.91 ± 4.26	40.38 ± 4.24	7.41 ± 0.89	8.80 ± 1.26
*MIX-J*	30	94.39 ± 1.10	15.15 ± 0.95	13.45 ± 0.68	20.54 ± 1.16
*TEJ*	92	95.03 ± 0.65	15.66 ± 0.63	14.35 ± 0.47	22.28 ± 0.70
*TRJ*	93	93.89 ± 0.74	15.78 ± 0.66	13.27 ± 0.39	20.57 ± 0.68
*TOL* *(MIX-J+TEJ+TRJ)*	215	94.45 ± 0.59	15.64 ± 0.39	13.76 ± 0.20	21.30 ± 0.36
*INT* *(ADMIX+ARO)*	22	80.41 ± 5.30	17.70 ± 0.36	11.93 ± 0.71	17.90 ± 1.28
*SEN* *(AUS+IND+MIX-I)*	133	21.16 ± 2.11	34.40 ± 1.13	6.76 ± 0.22	10.39 ± 0.29
*All*	370	67.27 ± 2.01	22.51 ± 0.66	11.13 ± 0.23	17.17 ± 0.36

**Table 2:** Summary of 20 *Multiple-Phenotype* (*MP*) QTL identified by GWAS mapping for four phenotyping assays of 370 RDP1 *O. sativa* accessions after a 7-day-10 °C chilling treatment. LTSS: Low Temperature Seedling Survivability; EL: Electrolyte Leakage; CAT: Catalase Activity; ANT: Anthocyanin Activity; LOD: Logarithm of the Odds; SNP: Single Nucleotide Polymorphism. See Supplementary Table S3 for details on published QTL.

QTL	Chr	Start	End	Phenotypes	Peak LOD Score	Peak SNP Gene	Published QTL
*qMP1-1*	1	8815194	9418864	CAT, LTSS	5.31	–	*qMT1-1* [40]
*qMP1-2*	1	18792455	18916961	ANT, CAT, LTSS	4.96	*LOC_Os01g34430*	*qMT1-4* [40], *qCTS1-2* [47]
*qMP1-3*	1	22290853	22489300	CAT, EL	4.27	*LOC_Os01g38740*	–
*qMP1-4*	1	37834095	37862889	CAT, EL	5.00	–	*qCTS1-4* [47]
*qMP3-1*	3	10104198	10284651	ANT, LTSS	5.46	–	*qLVG3* [46]
*qMP4-1*	4	2699247	2729293	CAT, EL	3.44	*LOC_Os04g05420*	*qCTS4-1* [47]
*qMP4-2*	4	4150962	4265645	ANT, EL	4.35	–	*qCTS4-1* [47]
*qMP4-3*	4	32076622	32401404	CAT, LTSS	6.19	–	*qMT4-3* [40], *qLTSS4-3* [37], *qLTG4* [46]
*qMP5-1*	5	3512674	4377698	ANT, CAT	3.45	–	*qLTG5-1* [46], *qCTS5-1* [47]
*qMP5-2*	5	23154623	23564490	CAT, EL	6.08	–	*qMT5-3* [40], *qCTSS-5* [46], *qCTS5-4* [47]
*qMP8-1*	8	3078138	3346129	ANT, CAT	4.21	–	*qMT8-1* [40], *qLTSS8-1* [37]
*qMP8-2*	8	21298422	21317944	ANT, LTSS	3.45	*LOC_Os08g34030*	*qMT8-3* [40], *qLTSS8-2* [37], *COLD2* [46], *qCTS8-4* [47]
*qMP8-3*	8	25620369	25655572	ANT, LTSS	6.22	–	*qMT8-4* [40]
*qMP10-1*	10	11675173	12345517	ANT, CAT	3.18	–	*qLTSS10-2* [37], *qCTSS-10* [46]
*qMP10-2*	10	13841307	14025821	ANT, LTSS	6.80	*LOC_Os10g26550*	*qMT10-4* [40], *qCTSS-10* [46]
*qMP11-1*	11	2685973	2766157	CAT, EL, LTSS	6.43	–	–
*qMP11-2*	11	28342262	28475216	ANT, EL	3.53	–	*qMT11-3* [40]
*qMP12-1*	12	2192895	2390059	ANT, LTSS	6.47	–	*qMT12* [40]
*qMP12-2*	12	8027010	8406160	CAT, EL	5.26	*LOC_Os12g14680*	*qCTSS-12* [46]
*qMP12-3*	12	9435619	9819447	CAT, EL	4.14	–	*qCTSS-12* [46]

## Data Availability

All data mentioned in this manuscript can be found in the Tables and Figures shown here and on the journal website as Supplementary Tables and Figures.

## Supplementary Materials

The following are available online at https://www.mdpi.com/article/10.3390/genes12111700/s1, Figure S1: Box plots of mean values for four cold tolerance response phenotypes after a 7-day-10 °C chilling treatment for two cold check accessions: *temperate japonica* Krasnodarskij 3352 (KR/blue; cold tolerant check) and *aus* Carolino-164 (CA/red; cold sensitive check), Figure S2: Quantile-Quantile (QQ) plots of GWAS mapping runs for four cold stress tolerance response phenotypes using 370 RDP1 accessions, Figure S3: Correlation analysis of two cold stress tolerance response phenotypes between this study (*x*-axis) and Shimoyama et al. 2020 [40] (*y*-axis), Figure S4: Distribution analysis of four cold stress tolerance response phenotypes for 370 RDP1 accessions, Figure S5: Distribution analysis of individual LTSS and EL activity phenotypes for three trials, Figure S6: Distribution analysis of individual CAT and ANT activity phenotypes for three trials, Figure S7: Box plots of mean values for CAT activity (A) and ANT activity (B) of 14-day-old seedlings for a subset of 70 RDP1 accessions grown under warm temperature (28 °C day/25 °C night) conditions, Table S1: Means, standard deviation, and variances of individual trials for four phenotyping assays using 370 RDP1 *O. sativa* accessions after a 7-day-10 °C chilling treatment, Table S2: Summary of 120 quantitative trait loci (QTL) identified by GWAS mapping for four cold stress tolerance response traits using 370 RDP1 accessions, Table S3: Positions of published QTL overlapping with the 20 *MP* QTL identified in this GWAS mapping study.
