# Supplementary material for: Low Temperature Antioxidant Activity QTL Associate with Genomic Regions Involved in Physiological Cold Stress Tolerance Responses in Rice (Oryza sativa L.)
Source: Genes (Basel). 2021 Oct 26;12(11):1700. doi: 10.3390/genes12111700 (PMC8618774; doi:10.3390/genes12111700)
Supplement: Supplementary file 1 [file genes-12-01700-s001.zip › genes-1409313-supplementary.pdf]

SUPPLEMENTARY FIGURES AND TABLES

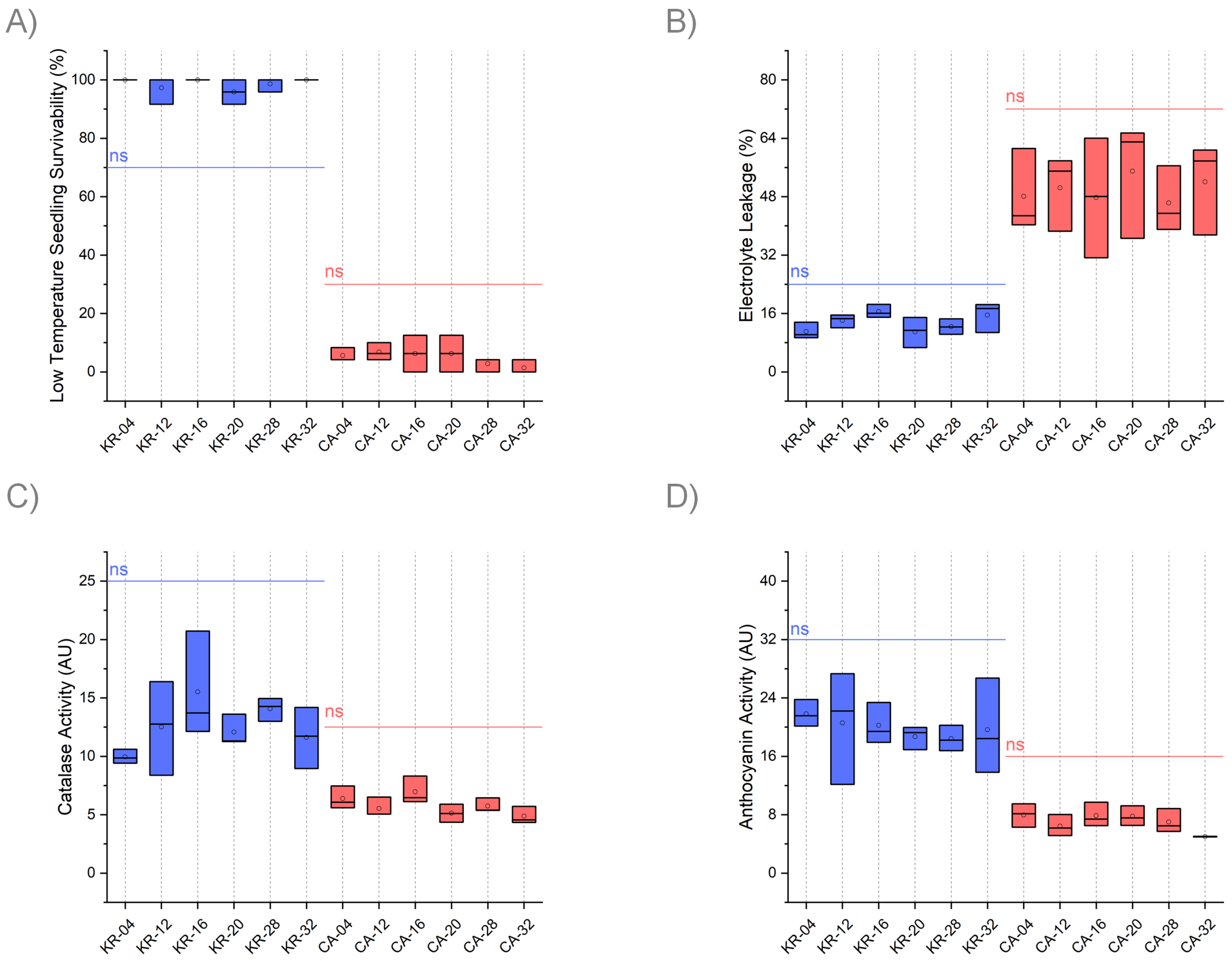

Supplementary Figure S1. Box plots of mean values for four cold tolerance response phenotypes after a 7-day-10°C chilling treatment for two cold tolerance check accessions: *temperate japonica* Krasnodarskij 3352 (KR/blue; cold tolerant check) and *aus* Carolino-164 (CA/red; cold sensitive check). (**A**) Low Temperature Seedling Survivability (LTSS) values for rice accessions of the three cold tolerance clusters. (**B**) Electrolyte Leakage (EL) values measuring membrane integrity after chilling stress for rice accessions of the three cold tolerance clusters. (**C**) Catalase (CAT) Activity values measuring enzymatic antioxidative activity for rice accessions of the three cold tolerance clusters. (**D**) Anthocyanin (ANT) Activity values measuring non-enzymatic antioxidative activity for rice accessions of the three cold tolerance clusters. Cold tolerance checks were grown in boxes of randomly selected sets (4, 12, 14, 20, 28, 32) as controls for every six sets of boxes (1-6; 7-12; 13-18; 19-24; 25-30; 31-32) out of 32 sets per experiment to show consistency in the environmental conditions.

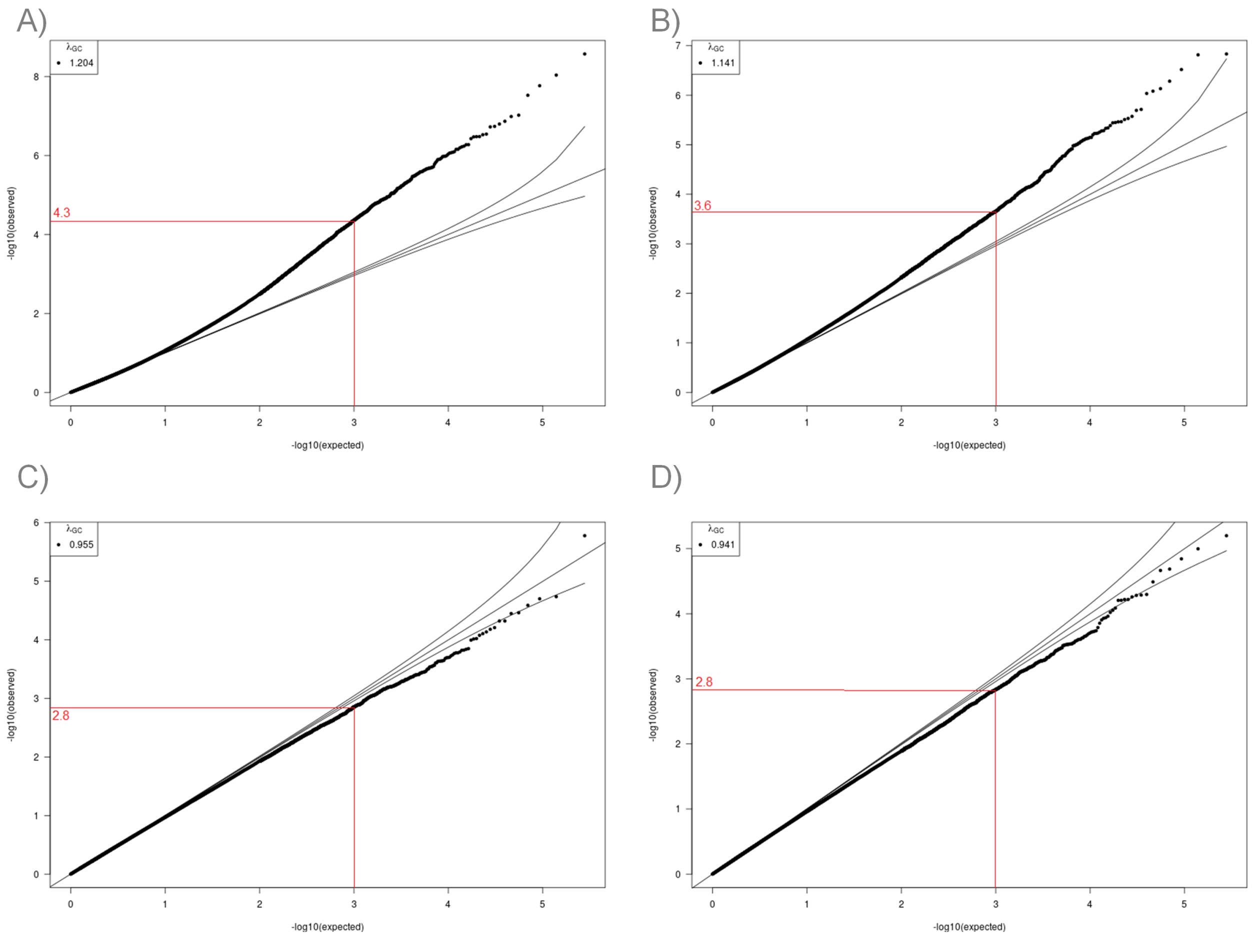

Supplementary Figure S2. Quantile-Quantile (QQ) plots of GWAS mapping runs for four cold stress tolerance response phenotypes using 370 RDP1 accessions. (**A**) Low Temperature Seedling Survivability (LTSS). (**B**) Electrolyte Leakage (EL). (**C**) Catalase Activity (CAT). (**D**) Anthocyanin Activity (ANT). Plots were created using the McCouch et al. 2016 RDP1 GWAS mapping pipeline [37]. Significance cutoff for SNPs meets the following requirements: -log10(*p*-expected)=3, and -log10(*p*-observed)=4.3 for LTSS; =3.6 for EL; and =2.8 for both CAT and ANT.

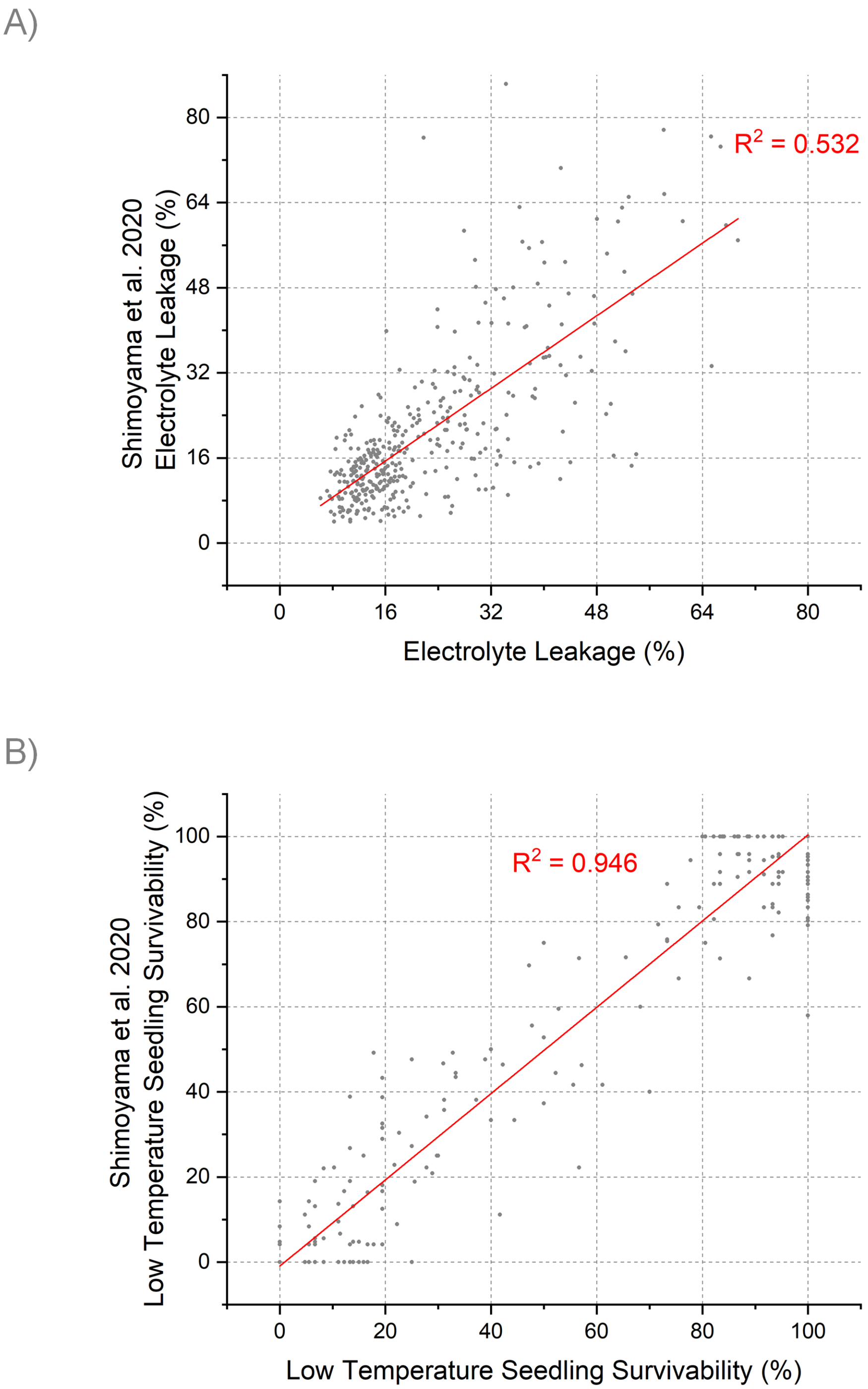

Supplementary Figure S3. Correlation analysis of two cold stress tolerance response phenotypes between this study (x-axis) and Shimoyama et al. 2020 [39] (y-axis). (**A**) Electrolyte Leakage (EL). (**B**) Low Temperature Seedling Survivability (LTSS). A linear fit is shown based on best-fit model calculations.

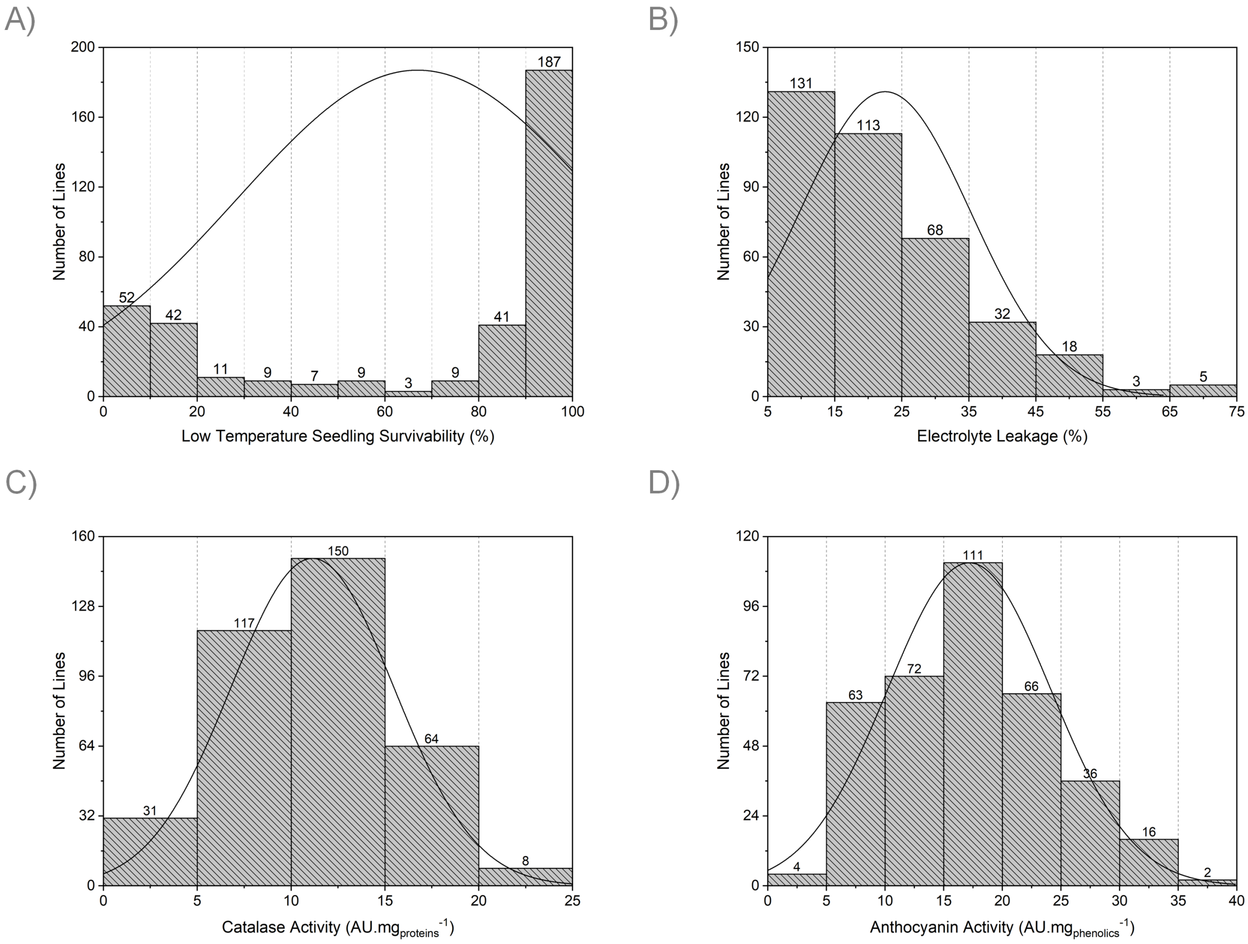

Supplementary Figure S4. Distribution analysis of four cold stress tolerance response phenotypes for 370 RDP1 accessions. (**A**) Low Temperature Seedling Survivability (LTSS). (**B**) Electrolyte Leakage (EL). (**C**) Catalase Activity. (**D**) Anthocyanin Activity. A normal distribution curve is calculated for each graph.

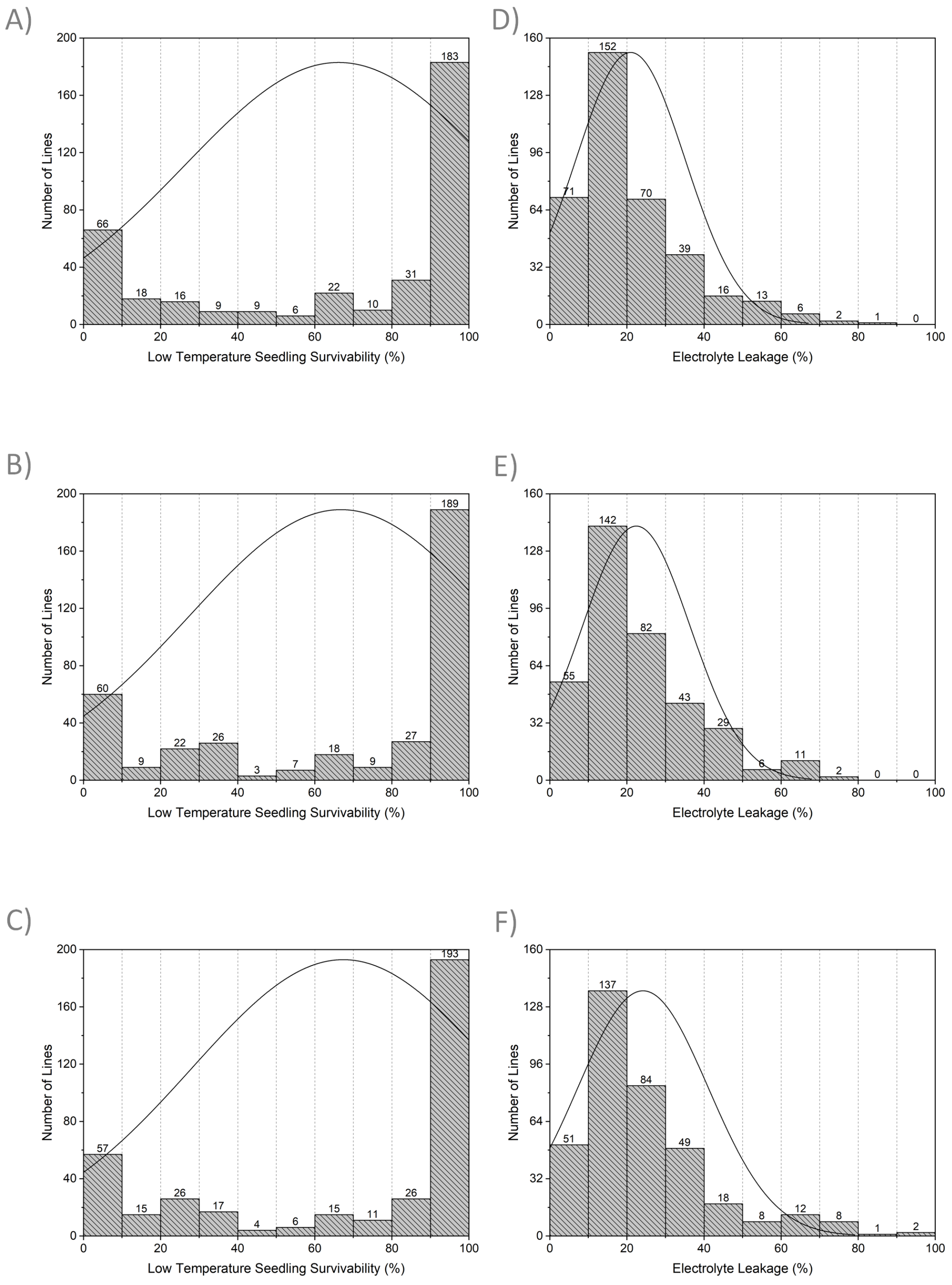

Supplementary Figure S5. Distribution analysis of individual LTSS and EL phenotypes for three trials. (**A**) LTSS experiment 1. (**B**) LTSS experiment 2. (**C**) LTSS experiment 3. (**D**) EL experiment 1. (**E**) EL experiment 2. (**F**) EL experiment 3.

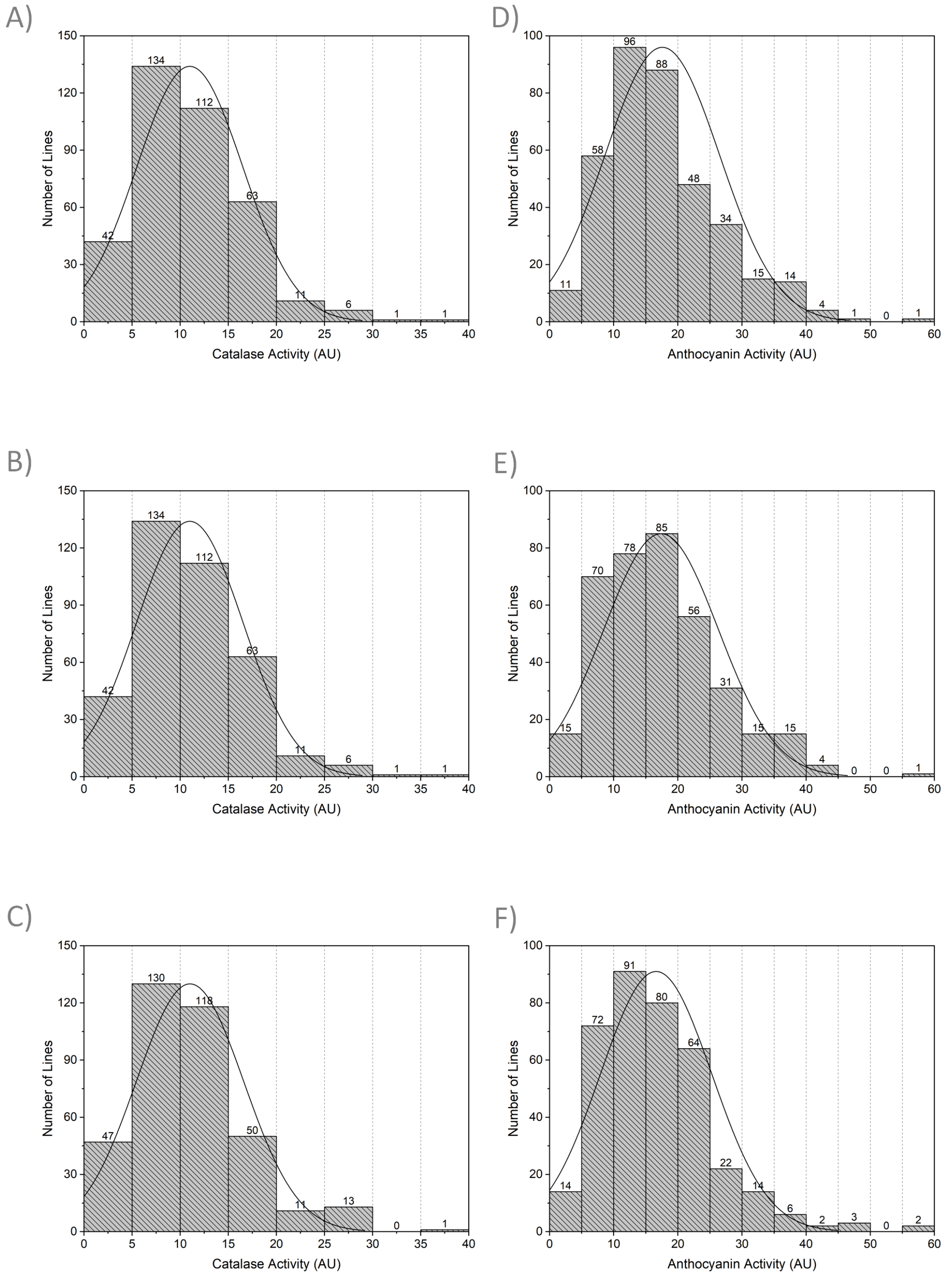

Supplementary Figure S6. Distribution analysis of individual CAT and ANT activity phenotypes for three trials. (**A**) CAT activity experiment 1. (**B**) CAT activity experiment 2. (**C**) CAT activity experiment 3. (**D**) ANT activity experiment 1. (**E**) ANT activity experiment 2. (**F**) ANT activity experiment 3.

A)

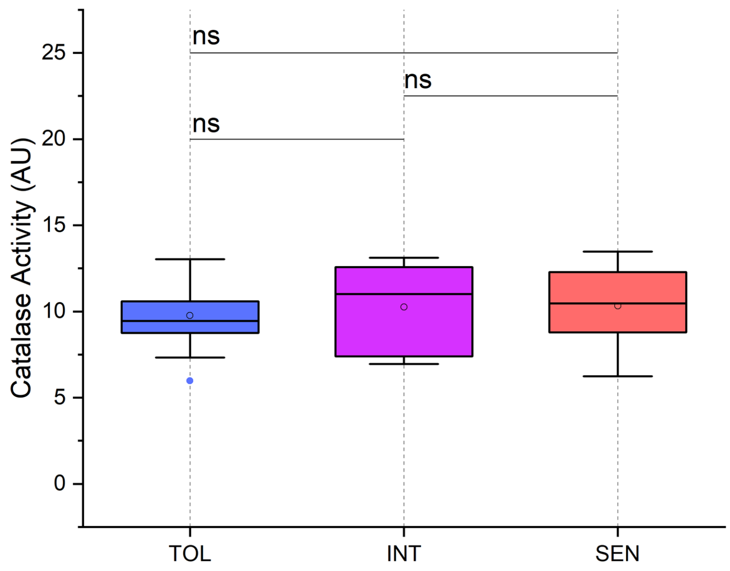

B)

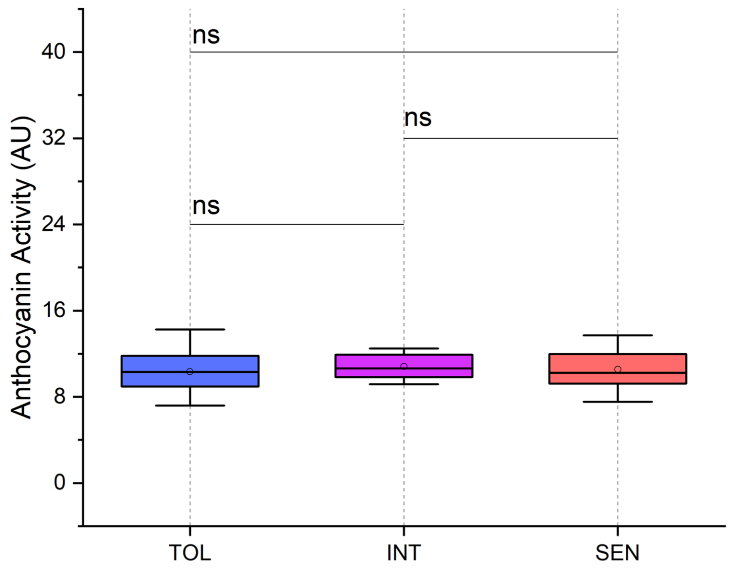

Supplementary Figure S7. Box plots of mean values for CAT activity (**A**) and ANT activity (**B**) of 14-day-old seedlings for a subset of 70 RDP1 accessions grown under warm control temperature (28°C day/25°C night) conditions.

Low Temperature Seedling Survivability (%)													
	No. of Cultivars	*Trial 1*			*Trial 2*			*Trial 3*			*Overall*		
		Mean	*Std. Dev.*	*Var.*	*Mean*	*Std. Dev.*	*Var.*	*Mean*	*Std. Dev.*	*Var.*	*Mean*	*Std. Dev.*	*Var.*
ADMIX	11	81.43	32.42	1051.02	81.36	23.58	556.01	77.88	31.88	1016.16	80.22	28.06	787.43
ARO	11	77.10	27.35	748.09	85.45	21.04	442.83	79.24	28.20	795.20	80.60	22.60	510.72
AUS	53	11.79	14.52	210.95	11.64	14.24	202.72	15.91	17.45	304.33	13.11	9.51	90.45
IND	74	27.85	34.11	1163.38	28.20	32.01	1024.56	26.26	31.65	1001.89	27.44	29.99	899.49
MIX-I	6	8.89	14.40	207.41	20.56	19.37	375.19	15.28	20.01	400.46	14.91	15.71	246.72
MIX-J	30	95.83	11.30	127.73	94.22	14.93	222.94	93.11	13.04	170.06	94.39	10.60	112.35
TEJ	92	95.09	11.04	121.85	93.24	12.88	165.90	96.76	7.88	62.11	95.03	7.36	54.11
TRJ	93	91.95	13.86	192.17	94.89	12.62	159.17	94.84	10.46	109.36	93.89	9.12	83.12
TOL (MIX-J + TEJ + TRJ)	215	93.83	12.43	154.52	94.09	13.03	169.77	95.42	9.91	98.14	94.45	8.62	74.25
INT (ADMIX + ARO)	22	79.26	29.35	861.63	83.41	21.91	480.02	78.56	29.38	863.04	80.41	24.86	618.20

SEN (AUS + IND + MIX-I)	133	20.59	28.31	801.22	21.25	26.92	724.89	21.64	26.76	716.12	21.16	24.34	592.29
All	370	66.64	40.37	1629.87	67.27	39.79	1583.06	67.90	39.77	1581.48	67.27	38.69	1497.05
**Electrolyte Leakage (%)**													
	No. of Cultivars	*Trial 1*			*Trial 2*			*Trial 3*			*Overall*		
		Mean	*Std. Dev.*	*Var.*	*Mean*	*Std. Dev.*	*Var.*	*Mean*	*Std. Dev.*	*Var.*	*Mean*	*Std. Dev.*	*Var.*
ADMIX	11	14.36	8.69	75.47	15.25	9.35	87.49	17.62	15.04	226.08	15.74	8.87	78.72
ARO	11	21.04	13.94	194.29	20.19	4.40	19.38	17.73	8.04	64.67	19.65	6.46	41.68
AUS	53	35.63	15.75	248.01	33.86	15.00	224.87	39.80	18.22	331.84	36.43	11.53	132.98
IND	74	29.52	16.35	267.18	32.47	14.90	222.11	35.43	20.20	408.15	32.47	14.09	198.52
MIX-I	6	32.70	8.24	67.91	42.04	6.16	37.95	46.41	17.34	300.59	40.38	6.24	38.97
MIX-J	30	14.17	6.43	41.33	14.29	7.19	51.64	17.01	9.19	84.54	15.15	6.13	37.56
TEJ	92	14.83	8.01	64.21	16.34	8.68	75.26	15.81	8.08	65.35	15.66	5.66	32.03
TRJ	93	13.96	6.76	45.72	16.39	9.48	89.84	16.99	8.25	68.10	15.78	5.58	31.18
TOL	215	14.36	7.26	52.73	16.08	8.84	78.14	16.48	8.30	68.86	15.64	5.67	142.18

(MIX-J + TEJ + TRJ)													
INT (ADMIX + ARO)	22	17.70	11.84	140.13	17.72	7.57	57.26	17.68	11.77	138.46	17.70	7.83	61.33
SEN (AUS + IND + MIX-I)	133	32.09	16.02	256.78	33.45	14.72	216.82	37.66	19.39	375.93	34.40	13.00	169.01
All	370	20.94	14.18	201.07	22.42	13.96	194.80	24.17	16.88	284.85	22.51	12.75	162.46
**Catalase Activity (AU)**													
	No. of Cultivars	*Trial 1*			*Trial 2*			*Trial 3*			*Overall*		
		Mean	*Std. Dev.*	*Var.*	*Mean*	*Std. Dev.*	*Var.*	*Mean*	*Std. Dev.*	*Var.*	*Mean*	*Std. Dev.*	*Var.*
ADMIX	11	10.15	3.31	10.98	13.13	6.14	37.72	12.4	4.33	18.72	11.89	2.99	8.97
ARO	11	12.99	6.09	37.07	9.85	3.58	12.84	13.05	4.77	22.73	11.96	3.79	14.37
AUS	53	6.64	2.89	8.32	6.71	2.79	7.79	6.70	3.48	12.10	6.68	2.39	5.72
IND	74	6.43	3.47	12.06	7.10	3.74	13.97	6.78	3.31	10.93	6.77	2.65	7.05
MIX-I	6	6.83	2.79	7.79	7.82	3.01	9.06	7.58	2.18	4.76	7.41	1.64	2.67
MIX-J	30	13.54	4.50	20.29	13.27	4.40	19.37	13.56	5.09	25.86	13.45	2.97	8.81

TEJ	92	14.63	5.18	26.79	14.69	4.94	24.4	13.73	5.5	30.3	14.35	2.82	7.96
TRJ	93	12.83	4.54	20.58	13.81	4.37	19.10	13.16	5.05	25.53	13.27	2.96	8.79
TOL (MIX-J + TEJ + TRJ)	215	13.70	4.87	23.70	14.11	4.64	21.49	13.46	5.24	27.44	13.76	2.94	8.62
INT (ADMIX + ARO)	22	11.57	5.00	25.00	11.49	5.19	26.91	12.72	4.46	19.85	11.93	3.33	11.11
SEN (AUS + IND + MIX-I)	133	6.53	3.20	10.26	6.98	3.35	11.21	6.78	3.32	11.03	6.76	2.50	6.27
All	370	11.00	5.50	30.30	11.39	5.42	29.33	11.02	5.58	31.09	11.13	4.34	18.81
**Anthocyanin Activity (AU)**													
	No. of Cultivars	*Trial 1*			*Trial 2*			*Trial 3*			*Overall*		
		Mean	*Std. Dev.*	*Var.*	*Mean*	*Std. Dev.*	*Var.*	*Mean*	*Std. Dev.*	*Var.*	*Mean*	*Std. Dev.*	*Var.*
ADMIX	11	14.09	4.81	23.17	16.12	6.83	46.58	15.76	6.22	38.63	15.33	4.42	19.55
ARO	11	21.55	7.57	57.31	18.03	8.87	78.69	21.84	6.41	41.04	20.47	6.48	41.96
AUS	53	10.7	4.77	22.71	9.99	3.79	14.35	10.2	4.27	18.23	10.3	3.04	9.26
IND	74	11.05	4.69	22.01	10.28	5.34	28.5	10.43	5.15	26.53	10.59	3.61	13

MIX-I	6	8.19	1.74	3.04	11.28	5.59	31.26	6.91	3.08	9.49	8.8	1.99	3.97
MIX-J	30	19.28	7.61	57.98	23.15	8.33	69.45	19.19	6.32	39.91	20.54	4.78	22.82
TEJ	92	23.08	9.04	81.77	22.61	8.25	68.03	21.14	8.25	68.02	22.28	5.47	29.97
TRJ	93	21.2	8.1	65.56	20.56	7.24	52.4	19.96	9.13	83.29	20.57	5.08	25.82
TOL (MIX-J + TEJ + TRJ)	215	21.74	8.52	72.56	21.8	7.88	62.07	20.36	8.41	70.67	21.3	5.26	27.66
INT (ADMIX + ARO)	22	17.82	7.27	52.92	17.08	7.78	60.6	18.8	6.9	47.61	17.9	6.02	36.23
SEN (AUS + IND + MIX-I)	133	10.78	4.65	21.58	10.21	4.76	22.67	10.18	4.77	22.74	10.39	3.34	11.13
All	370	17.57	8.92	79.53	17.35	8.8	77.47	16.61	8.67	75.19	17.17	6.97	48.6

Supplementary Table S1. Means, standard deviations, and variances of individual trials for four phenotyping assays using 370 RDP1 *O. sativa* accessions after a 7-day-10°C chilling treatment.

QTL	Chromosome	Start	End	Phenotype	Peak LOD Score	Peak SNP Gene
qANT1-1	1	7486614	8230878	ANT	3.54	–
qANT1-2	1	9773339	10350457	ANT	3.70	–
qANT1-3	1	12596561	13071111	ANT	3.10	*LOC_Os01g22954*<br>*LOC_Os01g22980*
qANT1-4	1	14626447	14810822	ANT	4.84	*LOC_Os01g25960*
qANT1-5	1	18651600	18916961	ANT	3.16	*LOC_Os01g34310*
qANT1-6	1	40773126	40950294	ANT	3.79	–
qANT2-1	2	19500004	20628603	ANT	3.05	–
qANT2-2	2	20789839	20971831	ANT	3.33	–
qANT2-3	2	25700171	25992998	ANT	3.18	–
qANT2-4	2	31287454	33240427	ANT	3.72	*LOC_Os02g54210*
qANT3-1	3	10104198	10986174	ANT	3.04	*LOC_Os03g19104*
qANT3-2	3	22831637	23578802	ANT	3.30	–
qANT4-1	4	3362640	3784630	ANT	3.72	*LOC_Os04g06790*
qANT4-2	4	4150962	4265645	ANT	2.99	*LOC_Os04g07830*
qANT4-3	4	20718231	20738002	ANT	4.21	–
qANT5-1	5	3021633	4377698	ANT	3.45	–
qANT5-2	5	25330495	26414180	ANT	3.53	–
qANT6-1	6	19661655	20361856	ANT	3.63	*LOC_Os06g34910*
qANT6-2	6	25767289	27349049	ANT	5.00	–
qANT7-1	7	4620300	4665149	ANT	3.05	LOC_Os07g08890
qANT7-2	7	7490073	8643262	ANT	3.58	LOC_Os07g15080
qANT8-1	8	2428291	3346129	ANT	4.20	–

qANT8-2	8	14193950	15347615	ANT	3.33	*LOC_Os08g23440*
qANT8-3	8	21298422	21317944	ANT	3.45	–
qANT8-4	8	25393379	26410436	ANT	4.69	–
qANT9-1	9	13119602	13800265	ANT	3.28	*LOC_Os09g23250*
qANT10-1	10	11675173	12345517	ANT	3.19	–
qANT10-2	10	12997744	14025821	ANT	4.02	–
qANT10-3	10	15461315	15790283	ANT	3.54	*LOC_Os10g30380*
qANT11-1	11	5570572	5826004	ANT	4.30	–
qANT11-2	11	24826908	26284095	ANT	4.22	–
qANT11-3	11	28342262	28475216	ANT	3.53	–
qANT12-1	12	902978	2390059	ANT	4.49	*LOC_Os12g02670*
qANT12-2	12	14551221	14974543	ANT	3.96	*LOC_Os12g25660*
qCAT1-1	1	8815238	9587843	CAT	3.13	–
qCAT1-2	1	10051543	10713488	CAT	3.70	*LOC_Os01g18950*
qCAT1-3	1	18219382	19606123	CAT	3.41	–
qCAT1-4	1	22290853	23591174	CAT	3.70	–
qCAT1-5	1	32077250	32971038	CAT	3.17	*LOC_Os01g55700*<br>*LOC_Os01g55690*
qCAT1-6	1	33508366	34917881	CAT	3.30	*LOC_Os01g60360*
qCAT1-7	1	37434095	38941678	CAT	3.21	*LOC_Os01g65200*
qCAT4-1	4	2699247	2729293	CAT	3.44	*LOC_Os04g05420*
qCAT4-2	4	27694251	28887295	CAT	3.32	*LOC_Os04g48370*
qCAT4-3	4	31662483	32401404	CAT	4.14	–
qCAT4-4	4	33478908	34926977	CAT	3.85	–
qCAT5-1	5	3512674	4775386	CAT	3.40	*LOC_Os05g07690*

qCAT5-2	5	8661995	9888457	CAT	3.64	−
qCAT5-3	5	14916027	15757372	CAT	5.78	−
qCAT5-4	5	17772273	18678752	CAT	3.37	−
qCAT5-5	5	22624937	23564490	CAT	3.74	−
qCAT6-1	6	7521403	8375233	CAT	3.17	−
qCAT6-2	6	23626779	24556297	CAT	4.00	−
qCAT7-1	7	15594791	15630559	CAT	3.49	*LOC_Os07g27000*
qCAT7-2	7	16129188	16960602	CAT	3.20	−
qCAT8-1	8	3078138	3557583	CAT	4.21	−
qCAT8-2	8	4686803	5100589	CAT	4.45	−
qCAT10-1	10	11565763	12409728	CAT	3.19	−
qCAT11-1	11	1956923	2809151	CAT	4.70	*LOC_Os11g04770*
qCAT11-2	11	10841118	10900649	CAT	3.26	*LOC_Os11g19030*
qCAT11-3	11	13056051	14038623	CAT	4.32	−
qCAT11-4	11	16085790	16938996	CAT	3.65	*LOC_Os11g27970*
qCAT11-5	11	17936031	18155767	CAT	3.22	*LOC_Os11g30790*
qCAT12-1	12	8027010	8888292	CAT	4.01	−
qCAT12-2	12	9435619	10107608	CAT	3.29	*LOC_Os12g17650*
qCAT12-3	12	22396394	22597096	CAT	3.51	*LOC_Os12g36860*
qEL1-1	1	5376814	6217016	EL	4.27	−
qEL1-2	1	21635429	22489300	EL	4.27	*LOC_Os01g38740*
qEL1-3	1	36919761	37862889	EL	4.77	−
qEL2-1	2	5367701	6404909	EL	5.20	−
qEL2-2	2	28049683	29312571	EL	6.04	−

qEL2-3	2	35713099	35777317	EL	4.40	*LOC_Os02g58400*
qEL3-1	3	4957753	5255713	EL	4.57	–
qEL3-2	3	14243353	15319978	EL	5.39	–
qEL3-3	3	21286603	21341380	EL	4.46	–
qEL3-4	3	30678991	30878159	EL	3.86	–
qEL4-1	4	209964	813396	EL	5.71	–
qEL4-2	4	2462372	2841611	EL	5.47	*LOC_Os04g05370*
qEL4-3	4	3426042	4453290	EL	4.73	*LOC_Os04g06700*
qEL4-4	4	5062444	5215299	EL	4.24	*LOC_Os04g09654*
qEL4-5	4	15936644	16045503	EL	4.67	–
qEL5-1	5	5533680	6761776	EL	4.71	–
qEL5-2	5	23154623	23812327	EL	6.08	–
qEL7-1	7	18672662	19698141	EL	5.33	–
qEL8-1	8	6312060	7733651	EL	4.22	–
qEL9-1	9	495867	1327074	EL	5.28	*LOC_Os09g02360*
qEL9-2	9	16868508	17684880	EL	6.13	*LOC_Os09g29110*
qEL10-1	10	19944099	20831382	EL	5.00	–
qEL11-1	11	2685973	2766157	EL	3.69	–
qEL11-2	11	14196428	14717817	EL	4.29	*LOC_Os11g24930*
qEL11-3	11	17425263	17627357	EL	5.53	*LOC_Os11g29980*
qEL11-4	11	18782766	19184008	EL	4.47	–
qEL11-5	11	22031752	22996111	EL	4.99	–
qEL11-6	11	27564332	28697913	EL	6.83	*LOC_Os11g45530*
qEL12-1	12	6958080	8406160	EL	5.26	*LOC_Os12g14680*

qEL12-2	12	9046310	9819447	EL	4.14	–
qLTSS1-1	1	3012508	3588139	LTSS	5.17	–
qLTSS1-2	1	6217016	7070844	LTSS	6.08	–
qLTSS1-3	1	8815194	9418864	LTSS	5.31	–
qLTSS1-4	1	18792455	18989383	LTSS	4.96	*LOC_Os01g34430*
qLTSS3-1	3	8169374	8209158	LTSS	7.77	*LOC_Os03g14980*
qLTSS3-2	3	10032950	10284651	LTSS	5.46	–
qLTSS3-3	3	21124409	22179741	LTSS	5.22	–
qLTSS3-4	3	24553451	24646868	LTSS	5.66	–
qLTSS4-1	4	18188624	19063931	LTSS	5.24	–
qLTSS4-2	4	32076622	32422788	LTSS	7.53	–
qLTSS5-1	5	20728233	21496273	LTSS	6.10	*LOC_Os05g35140*
qLTSS6-1	6	4696360	4973808	LTSS	6.52	–
qLTSS6-2	6	11948492	12073609	LTSS	6.07	–
qLTSS8-1	8	16343497	17184778	LTSS	4.95	–
qLTSS8-2	8	18000666	18923306	LTSS	5.90	*LOC_Os08g30680*
qLTSS8-3	8	21253044	22052077	LTSS	5.27	–
qLTSS8-4	8	25620369	25655572	LTSS	6.22	–
qLTSS9-1	9	16351083	17147264	LTSS	7.02	*LOC_Os09g27710*
qLTSS10-1	10	1789478	2402873	LTSS	5.89	–
qLTSS10-2	10	5282251	5324028	LTSS	5.53	–
qLTSS10-3	10	13841307	14851265	LTSS	6.99	*LOC_Os10g27360*
qLTSS10-4	10	21081923	22064791	LTSS	4.97	*LOC_Os10g40612*
qLTSS11-1	11	2073424	2766157	LTSS	6.43	–

qLTSS12-1	12	2192895	3113518	LTSS	6.47	*LOC_Os12g05040*
qLTSS12-2	12	22607831	22668332	LTSS	6.48	*LOC_Os12g36890*

Supplementary Table S2. Summary of 120 quantitative trait loci (QTL) identified by GWAS mapping for four cold stress tolerance response traits using 370 RDP1 accessions. LTSS: Low Temperature Seedling Survivability; EL: Electrolyte Leakage; CAT: Catalase Activity; ANT: Anthocyanin Activity; LOD: Logarithm of the Odds; SNP: Single Nucleotide Polymorphism.

QTL	Chromosome	Location	Phenotype(s)	Publication
qMT1-1	1	10527625 – 11389874	Electrolyte Leakage at 10°C<br>Low Temperature Seedling Survivability at 10°C	Shimoyama et al. 2020
qMT1-4	1	19518259 – 20986910	Electrolyte Leakage at 8°C<br>Electrolyte Leakage at 12°C	Shimoyama et al. 2020
qCTS1-2	1	18680885 – 19680885	Cold Tolerance Score at 8°C	Wang et al. 2016
qCTS1-4	1	40885263 – 41885263	Cold Tolerance Score at 8°C	Wang et al. 2016
qLVG3	3	~ 9500000 – 12700000	Electrolyte Leakage at 4°C<br>Electrolyte Leakage at 5-12°C	Lv et al. 2016
qCTS4-1	4	5350082 – 6350082	Cold Tolerance Score at 8°C	Wang et al. 2016
qMT4-3	4	34467694 – 35089971	Electrolyte Leakage at 4°C<br>Low Temperature Seedling Survivability at 10°C	Shimoyama et al. 2020
qLTSS4-3	4	~ 34200000 – 35200000	Low Temperature Seedling Survivability at 10°C	Schläppi et al. 2017
qLTG-4	4	~ 30000000 – 33000000	Electrolyte Leakage at 4°C<br>Electrolyte Leakage at 5-12°C	Lv et al. 2016
qLTG5-1	5	~ 4300000 – 7400000	Electrolyte Leakage at 4°C<br>Electrolyte Leakage at 5-12°C	Lv et al. 2016
qCTS5-1	5	1470193 – 2470193	Cold Tolerance Score at 8°C	Wang et al. 2016
qMP5-2	5	6600746 – 8783967	Electrolyte Leakage at 10°C<br>Low Temperature Seedling Survivability at 10°C<br>Low Temperature Seedling Survivability at 12°C	Shimoyama et al. 2020
qCTSS-5	5	~ 25600000 – 28000000	Electrolyte Leakage at 4°C<br>Electrolyte Leakage at 5-12°C	Lv et al. 2016
qCTS5-4	5	24116513 – 34116513	Cold Tolerance Score at 8°C	Wang et al. 2016

qMT8-1	8	4409763 – 5060164	Electrolyte Leakage at 12°C<br>Low Temperature Seedling Survivability at 12°C	Shimoyama et al. 2020
qLTSS8-1	8	~ 5200000 – 6200000	Low Temperature Seedling Survivability at 10°C	Schläppi et al. 2017
qMT8-3	8	16369660 – 16896911	Low Temperature Seedling Survivability at 4°C<br>Low Temperature Seedling Survivability at 12°C	Shimoyama et al. 2020
qLTSS8-2	8	~ 14900000 – 15900000	Low Temperature Seedling Survivability at 10°C	Schläppi et al. 2017
COLD2	8	~ 22900000 – 25600000	Electrolyte Leakage at 4°C<br>Electrolyte Leakage at 5-12°C	Lv et al. 2016
qCTS8-4	8	19885371 – 20885371	Cold Tolerance Score at 8°C	Wang et al. 2016
qMT8-4	8	26014290 – 27371604	Electrolyte Leakage at 8°C<br>Electrolyte Leakage at 10°C<br>Low Temperature Seedling Survivability at 16°C	Shimoyama et al. 2020
qLTSS10-2	10	~ 11100000 – 12100000	Low Temperature Seedling Survivability at 10°C	Schläppi et al. 2017
qCTSS-10	10	~ 12000000 – 16000000	Electrolyte Leakage at 4°C<br>Electrolyte Leakage at 5-12°C	Lv et al. 2016
qMT10-4	10	12242058 – 15899908	Low Temperature Seedling Survivability at 10°C<br>Low Temperature Seedling Survivability at 12°C	Shimoyama et al. 2020
qMT11-3	11	25159572 – 25809234	Low Temperature Seedling Survivability at 10°C<br>Low Temperature Seedling Survivability at 12°C	Shimoyama et al. 2020
qMT12	12	2192895 – 4043986	Electrolyte Leakage at 8°C<br>Low Temperature Seedling Survivability at 10°C<br>Low Temperature Seedling Survivability at 12°C	Shimoyama et al. 2020
qCTSS-12	12	~ 5500000 – 9600000	Electrolyte Leakage at 4°C<br>Electrolyte Leakage at 5-12°C	Lv et al. 2016

Supplementary Table 3. Positions of published QTL overlapping with the 20 *MP* QTL identified in this GWAS mapping study.
